# Characterization of a Rice GH5_11 Gene Associated with Endosperm and Seed Traits

**DOI:** 10.3390/plants14223428

**Published:** 2025-11-09

**Authors:** Koen Gistelinck, Zoë Madder, Isabel Verbeke, Els J. M. Van Damme

**Affiliations:** Laboratory of Biochemistry and Glycobiology, Department of Biotechnology, Faculty of Bioscience Engineering, Ghent University, 9000 Ghent, Belgium; koen.gistelinck@ugent.be (K.G.); zoe.madder@ugent.be (Z.M.); isabel.verbeke@ugent.be (I.V.)

**Keywords:** glycosyl hydrolase, rice, chalkiness, fertility, cell wall

## Abstract

The plant cell wall is essential for maintaining cellular structure and regulating physiological processes such as growth and stress tolerance. Cell wall dynamics are largely mediated by cell wall-modifying enzymes, including glycoside hydrolases (GHs). In this study, we explored GH5 family members in *Oryza sativa* L. and identified 17 genes encoding GH5 proteins, classified into three subfamilies: GH5_7, GH5_11, and GH5_14. Characterization of the GH5_11 protein encoded by the *LOC_Os04g40510* gene involved the subcellular localization of a GFP-tagged protein, gene expression analysis during germination, and phenotypic evaluation of transgenic plants. The protein was synthesized through the secretory pathway with expression in seeds, predominantly in the endosperm. Overexpression of *LOC_Os04g40510* resulted in altered seed morphology, increased chalkiness, and reduced seed set. Although the overall seed number increased, the seed mass was reduced for the knock-down lines. These data suggest that *LOC_Os04g40510* may play a role in fertility and endosperm development. Our findings provide new insights into the biological function of GH5_11 enzymes in rice.

## 1. Introduction

Cereals are a primary food source for humans all over the world. Wheat, rice, and maize account for approximately 90% of the global production, serving as staple foods for billions of people [1,2]. Rice (*Oryza sativa* L.) is cultivated on every continent except Antarctica, providing nourishment for more than half of the world’s population [3,4]. The importance of rice in food security and its adaptability to diverse environments make this crop a good target for yield improvement. However, enhancing rice performance under various growth conditions requires a good understanding of the underlying molecular processes.

The cell wall and the enzymes involved in its biosynthesis and remodeling significantly contribute to several physiological processes [5,6]. Cell wall components—including cellulose, hemicellulose, pectins, and structural proteins—contribute to plant architecture, mechanical strength, and stress resilience, which in turn influence overall crop performance and productivity in rice [7,8,9,10,11,12,13,14,15,16,17]. Consequently, characterizing the molecular players involved in rice cell wall modification is an important target for improving agronomic traits and stress tolerance. For instance, chalkiness in rice has been associated with plant cell wall dynamics [18,19,20,21].

Rice endosperm is generally translucent but, in some cases, opaque or white patterns are observed in the kernel, which is termed chalkiness; an undesired feature given that these seeds are more prone to breaking during milling, resulting in yield loss and reduced consumer acceptability [18,22]. Scanning electron microscopy revealed that chalky endosperm consists of loosely packed starch granules with small air pockets, compared to the translucent endosperm which is densely packed [19,23]. The cause of chalkiness is complex and is determined by multiple factors. This grain feature is correlated with enhanced starch breakdown and cell wall decomposition [20,21]. In a recent review paper, Chen et al. (2024) provide an overview of the phenomena causing chalkiness [21].

The breakdown of glycosidic linkages in cell wall polysaccharides is mediated by glycosyl hydrolases (GHs). This group of enzymes can be classified based on amino acid sequence [24,25,26]. Approximately 40 distinct glycosyl hydrolase families have been identified in the rice genome [27]. The GH5 family is one of the largest glycoside hydrolase (GH) families and ranks among the top 10 most abundant GH families in *O. sativa* L. [27,28]. Despite the abundance, the functional roles of most GH5 members in plants remain poorly understood. The identified GH5 enzymes in *Oryza sativa* L. are part of the GH5_7, GH5_11, and GH5_14 subfamilies. Interestingly, these subfamilies are associated with distinct enzymatic activities [27,29]. GH5_7 enzymes are typically characterized by endo-β-1,4-mannanase activity, while GH5_14 enzymes are associated with exo-β-1,3- and exo-β-1,4-glucosidase activity. To date, no GH5_11 enzymes have been characterized. Although GH5_11 enzymes are often suggested to have either endo-β-1,4-mannanase or endo-β-1,4-glucanase activity [30,31], biochemical data are currently lacking.

Information on the biochemical properties and biological functions of rice GH5 proteins is limited, with only few reports available. For example, knock-out of the *Low Seed Setting Rate 1* (*LSSR1*) gene (*LOC_Os02g38260*), which encodes a member of the GH5_11 subfamily, significantly reduced the seed setting rate due to impaired pollen tube guidance toward the ovules [17]. Although LSSR1 has been annotated as a putative cellulase; it has not yet been characterized at biochemical level. Another example is *OsGH5BG* (*LOC_Os10g22520*), a member of the GH5_14 subfamily. This gene is highly expressed in the shoot during germination and in the leaf sheaths of mature plants. Its expression is also upregulated in response to various abiotic stresses and plant hormones, including salt, submergence, methyl jasmonate, and abscisic acid. The recombinant enzyme of OsGH5BG hydrolyzed several *p*-nitrophenyl (*p*NP) glycosides, including *p*NP-β-d-fucoside, *p*NP-β-d-glucoside, and *p*NP-α-l-arabinoside. Additionally, OsGH5BG showed activity against β-(1,4)-linked glucose oligosaccharides and the β-(1,3)-linked disaccharide laminaribiose [32].

GH domains are commonly linked to auxiliary modules such as carbohydrate-binding/lectin-like domains, which often enhance substrate affinity or catalytic efficiency [33]. A previous study on lectin-domain-containing proteins identified three members of the GH5_11 subfamily that possess a ricin B-like domain [34]. Among these, *LOC_Os04g40510* is the closest homolog of *LSSR1*, a gene implicated in seed setting and pollen fertility [17]. Given this phylogenetic relationship and the peculiar domain composition, *LOC_Os04g40510* was selected for characterization to investigate whether it may perform a similar or related biological function. To date, no biochemical characterization or detailed biological function has been reported for *LOC_Os04g40510*. Transcriptomic studies have suggested a possible involvement of this gene in seed development and grain quality. For example, knock-out of the transcription factor *OsNAC02* resulted in chalky seeds, and *LOC_Os04g40510* was identified as a differentially expressed gene showing reduced transcript levels in the mutant [35]. Conversely, when evaluating seed milling quality, several rice accessions with high milling performance (high head milling rate) displayed lower expression of *LOC_Os04g40510* during the grain-filling stage [36]. These observations point to a potential role of *LOC_Os04g40510* in seed development.

In this study, the *LOC_Os04g40510* gene from rice encoding a GH5_11 enzyme, a paralog of *LSSR1*, was selected for investigation [17,27]. To date, little is known about the gene product of *LOC_Os04g40510*. To gain more insight into the biological role of *LOC_Os04g40510* in plants, we investigated gene expression in early developmental stages, promoter activity, subcellular localization of the protein, and phenotypic effects in transgenic lines with altered expression of this enzyme. The results highlight that the GH5_11 enzyme plays a significant role in several rice seed traits and developmental processes.

## 2. Results

### 2.1. GH5 Sequences Within the Rice Genome

Amino acid sequences encoding GH5 enzymes were identified in rice using the Phytozome database and the corresponding InterPro identifier. In total, 17 different genes were retrieved. Although four of these genes contained splice variants, only two, namely *LOC_Os01g47400* and *LOC_Os03g61280*, had splice variants that resulted in distinct amino acid sequences. GH subfamily classification predicted multiple GH5 subfamilies, namely GH5_7 (*n* = 9), GH5_11 (*n* = 4), and GH5_14 (*n* = 4).

The distribution of the GH5 genes was mapped onto the rice genome (Supplementary Figure S1A), showing that the distinct genes are distributed across the rice chromosomes, though no GH5 genes were identified on chromosomes 7 and 9. Analysis of duplication events (Supplementary Figure S1B) revealed several clusters corresponding to the distinct GH5 subfamilies, with no apparent duplication links between subfamilies. The largest cluster contains the GH5_7 members, and gene duplication occurred primarily by dispersed duplications and, more specifically, through DNA-transposed duplication. In the case of the GH5_11 cluster, diverse types of duplication mechanisms resulted in the current diversity within the rice genome such as dispersed, segmental, and tandem duplications. For the cluster with GH5_14 genes, the duplication events were less interconnected compared to the GH5_7 and GH5_11 clusters. The correlated types of duplication were dispersed duplication, for which only one was defined as a DNA-transposed duplication event, and proximal duplication, which occurred for the *LOC_Os10g22570-LOC_Os10g22520* gene pair.

**Figure 1:**
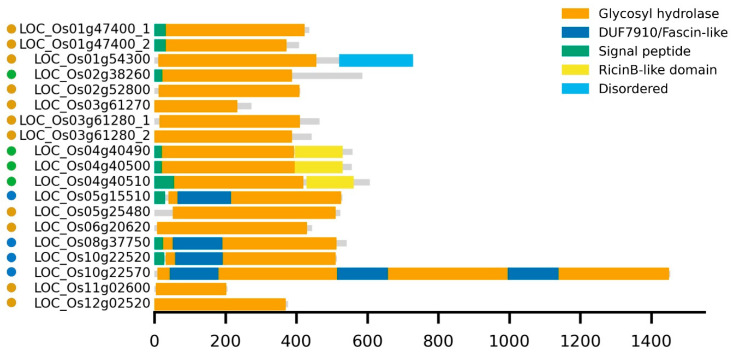
Schematic representation of predicted domain architectures for the GH5 proteins. Each horizontal gray bar represents the full-length protein (to scale), with colored blocks indicating annotated domains. Domain positions and sizes are shown relative to the total amino acid length for each protein. Amino acid positions are indicated on the x-axis. Yellow, green, and blue dots, next to the protein names, correspond to members from the GH5_7, GH5_11, and GH5_14 subfamilies, respectively.

Domain modularity was assessed for the distinct proteins (Figure 1). All GH5_7 sequences comprise a single GH5 domain. Only the LOC_Os01g47400 variants contain a signal peptide, and *LOC_Os01g54300* encodes a protein with a disordered region at the *C-*terminus. Members of the other subfamilies contain signal peptides (except for LOC_Os10g22570) and possess additional domains. The GH5_11 enzymes contain a ricin B-like lectin domain, except for LOC_Os02g38260 (also known as LSSR1). However, there is a *C*-terminal peptide of at least 100 amino acids in this protein. GH5_14 members contain a ‘Domain of Unknown Function (DUF) 7910’ or Fascin-like domain (within the TIM barrel structure of the GH5 domain). The largest protein, LOC_Os10g22570, was part of the GH5_14 subfamily and contains three copies of the DUF7910-glycosyl hydrolase unit.

**Figure 2:**
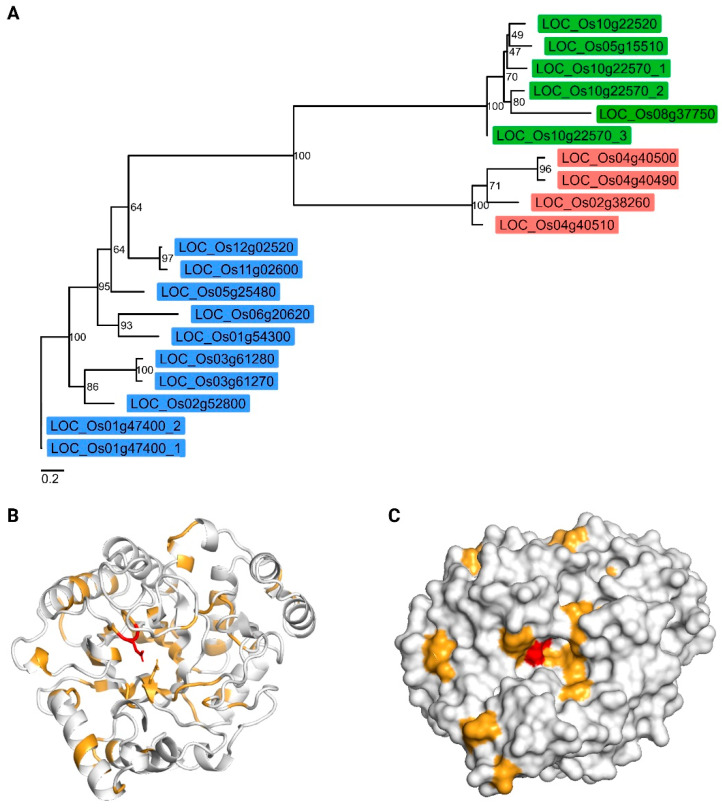
Comparative phylogenetic and structural analysis of rice GH5 domains. (**A**) Maximum likelihood phylogenetic tree constructed using the GH5 domain sequences from *Oryza sativa* L. to examine the evolutionary relationships within the gene family. Sequences consisted solely of the GH5 domain. The tree was inferred using IQ-TREE2 under the WAG+I+G4 model, with node support assessed via 1000 ultrafast bootstrap replicates and SH-aLRT. Tip labels represent gene names. Branch lengths are proportional to evolutionary distance (reference bottom left). The label colors represent the distinct subfamilies. Green, red, and blue represent GH5_14, GH5_11, and GH5_7, respectively. (**B**) Predicted three-dimensional structure of the GH5 domain of LOC_Os04g40510 shown in cartoon representation, with catalytic glutamate residues displayed as sticks. (**C**) Surface representation of the same GH5 domain model, highlighting the catalytic pocket in the same orientation as in panel B. For both panels (**B**,**C**), identical residues are colored in red and chemically equivalent residues in orange, as defined by the ESPript analysis based on the multiple sequence alignment (Supplementary Figure S2).

The amino acid sequences of all GH5 domains from *O. sativa* L. were used to construct a maximum likelihood phylogenetic unrooted tree (Figure 2A). It is clear that the GH5 members are clustered based on subfamilies. It should be noted that the average evolutionary distance between the distinct subfamilies is very similar. The average pairwise evolutionary distance (±standard deviation) between subfamily GH5_7 and GH5_11 was 4.19 ± 0.376. In the case of GH5_7 and GH5_14, the average evolutionary distance is 4.26 ± 0.402. The average evolutionary distance between GH5_11 and GH5_14 is 4.06 ± 0.354.

Multiple sequence alignment (Supplementary Figure S2) revealed that several sequences lacked a complete GH5 domain, in particular LOC_Os01g47400_2, LOC_Os03g61270, and LOC_Os11g02600. The alignment revealed conservation of only two residues across the rice GH5 sequences: an asparagine immediately upstream of the first catalytic glutamate. The catalytic glutamate residues were conserved in most sequences; however, LOC_Os11g02600 and LOC_Os01g47400_2 lacked the second glutamate important for catalytic activity. Mapping the residue conservation pattern onto the three-dimensional structure of LOC_Os04g40510 highlighted that only a few residues were located within the catalytic site, suggesting functional divergence and potential diversification of substrate specificity among GH5 family members (Figure 2B,C).

### 2.2. Expression Analysis for LOC_Os04g40510 Gene

Data from the Expression Atlas revealed that the *LOC_Os04g40510* gene is expressed across specific tissues and developmental stages, with the highest transcript levels observed in the seeds and predominantly in the endosperm (Supplementary Figure S3).

**Figure 3:**
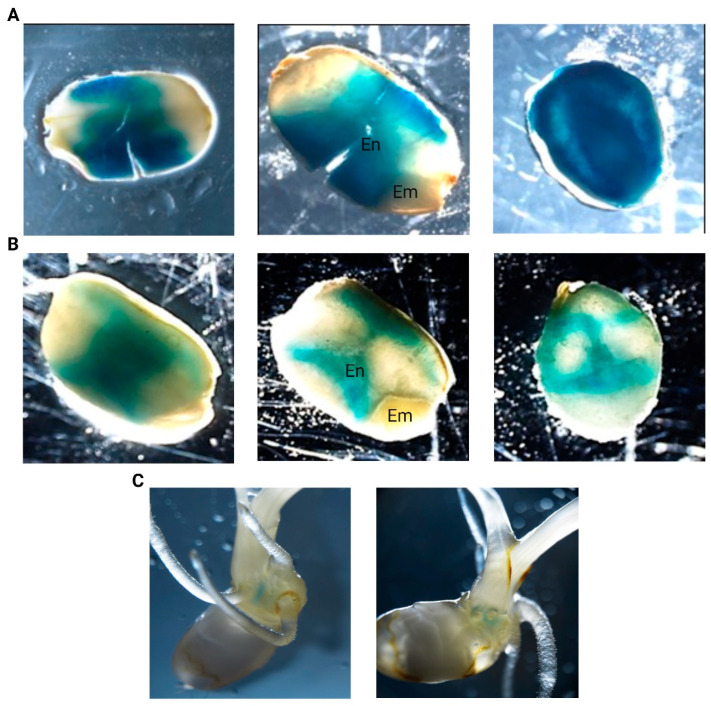
GUS histochemical analysis of pLOC_Os04g40510::GUS transgenic rice seeds and seedlings. GUS staining patterns in (**A**) dry, (**B**) imbibed seeds, and (**C**) 7 DPI-old seedlings are shown. Several images were taken from the seeds including non-sectioned seeds (first column), longitudinal sections (second column), and transverse sections (third column). Endosperm and embryo were indicated with ‘En’ and ‘Em’, respectively.

GUS histochemical staining assays with the GUS reporter rice lines containing the promoter sequence of *LOC_Os04g40510* in tandem with the GUS reporter gene enabled to check for promotor activity in various tissues and developmental stages. GUS staining was consistently observed in the seeds of pLOC_Os04g40510::GUS transgenic (T3) lines (Figure 3). GUS staining was observed in dry seeds, in imbibed seeds, and in the scutellum at later developmental stages (7 days post-imbibition (DPI)). Dry seeds displayed high promoter activity, whereas imbibition reduced the intensity of the GUS staining drastically, suggesting that the promotor activity decreases after imbibition. Moreover, the GUS staining displayed distinct spatial patterns in the endosperm while the embryo was not stained. No promoter activity was detected in the vegetative tissues after germination or during flowering.

**Figure 4:**
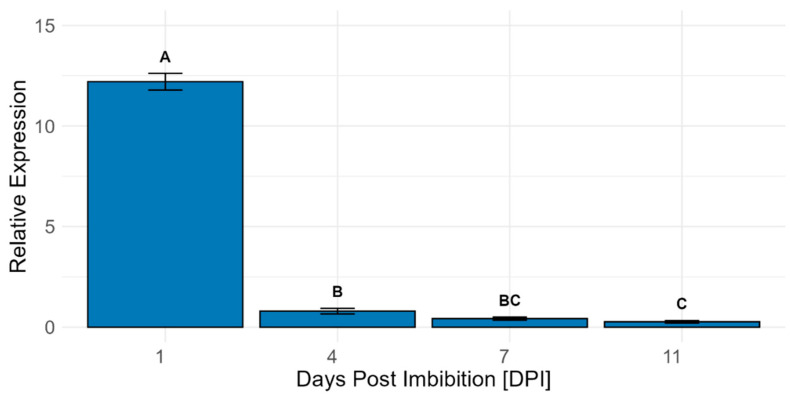
Relative expression values for *LOC_Os04g40510* in whole seedlings during early development. Whole seedlings (including roots, shoots, and seed) from 10 wild-type seedlings, grown on agar plates, were pooled as one biological replicate. For each time point, three biological replicates were used. Expression was analyzed using a one-way ANOVA followed by multiple hypothesis correction using the Tukey–Kramer method. The error bars represent the standard error of the samples, and the letters indicate the significant differences between the tissues at the *p* < 0.05 level.

The transcript levels for the gene of interest were quantified in early developmental stages by RT-qPCR analysis (Figure 4). The relative expression of *LOC_Os04g40510* was very high after one day of imbibition (DPI) but was almost completely abolished after 4 DPI.

### 2.3. Transient Expression of LOC_Os04g40510 in N. benthamiana Leaf Epidermal Cells

**Figure 5:**
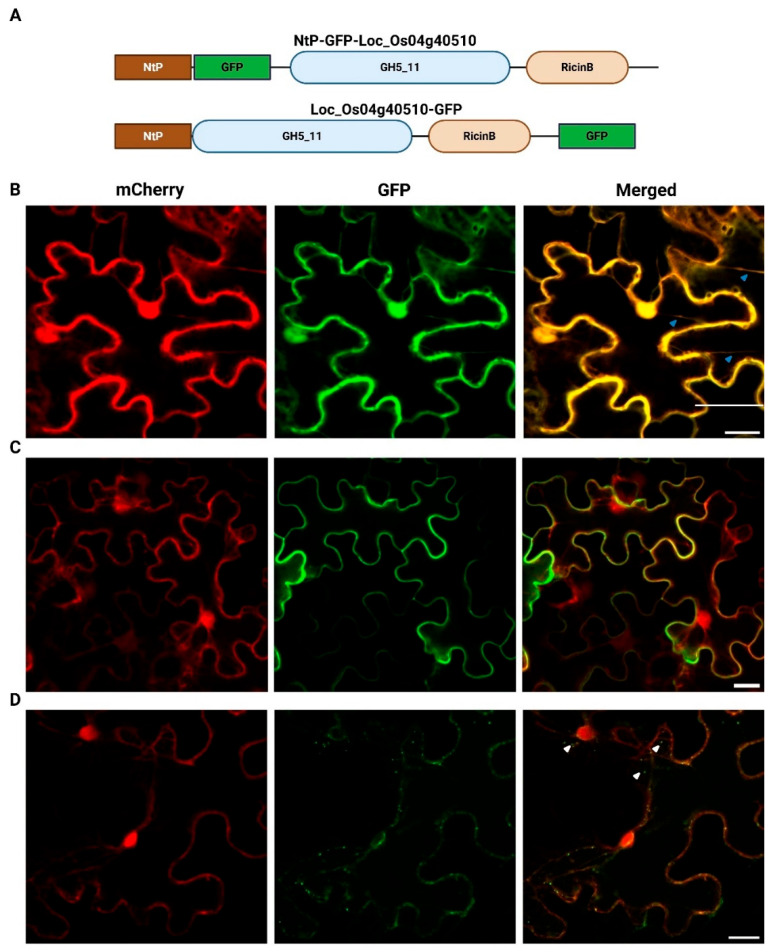
(**A**) Schematic overview of the constructs and the confocal fluorescence images of *N. benthamiana* leaf epidermal cells co-infiltrated with mCherry in combination with (**B**) free eGFP, (**C**) NtP-GFP-LOC_Os04g40510 or (**D**) LOC_Os04g40510-GFP. Merged image (third column) shows co-localization of mCherry (first column), GFP constructs (second column). Blue and white arrowheads were used to indicate cytoplasmic strands and vesicle-like structures, respectively. Images were acquired using Nikon A1R confocal microscope. The scale bar corresponds to 20 µm. “NtP” refers to the endogenous *N*-terminal signal peptide predicted for *LOC_Os04g40510*. Schematic overview was generated using BioRender.

The localization of the LOC_Os04g40510 protein was determined using two distinct GFP-fusion proteins (Figure 5), a GFP insertion construct in which GFP is localized after the *N-*terminal signal peptide (NtP-GFP-LOC_Os04g40510) and a *C*-terminal GFP fusion (LOC_Os04g40510-GFP) (Figure 5A). Tobacco leaf cells co-infiltrated with both mCherry and free eGFP constructs served as a control. In these cells, free eGFP and mCherry co-localize in the cytoplasm and the nucleus, as shown by the presence of the cytoplasmic strands and nuclear signals (Figure 5B). The NtP-GFP-LOC_Os04g40510 protein and the *C*-terminally GFP-tagged LOC_Os04g40510 fusion protein do not localize to the cell nucleus, but fluorescence is observed in the cytoplasm, near the cell membrane, and cell wall. The NtP-GFP-LOC_Os04g40510 fusion protein displays a uniform localization along the cell periphery (Figure 5C). In contrast, the LOC_Os04g40510-GFP fusion protein shows an uneven distribution at the cell periphery and accumulates in vesicle-like structures within the cytoplasm and around the cell nucleus (Figure 5D). Variation in fluorescent signal intensity between constructs was detected, and this pattern was consistently reproduced in at least three independent infiltration experiments.

**Figure 6:**
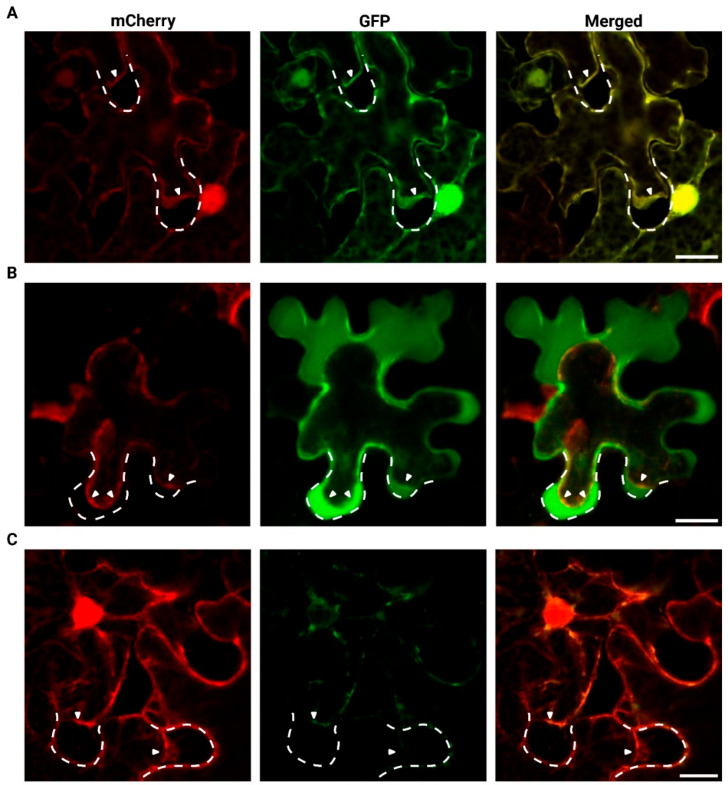
Confocal fluorescence images of *N. benthamiana* leaf epidermal cells co-infiltrated with mCherry in combination with (**A**) free eGFP, (**B**) NtP-GFP-LOC_Os04g40510 or (**C**) LOC_Os04g40510-GFP after treatment with 5 M NaCl for maximum 2 min. Merged image (third column) shows co-localization of mCherry (first column) and GFP constructs (second column). Images were acquired using Nikon A1R confocal microscope. The length of the scale bar corresponds to 20 µm. The dashed white lines show the original position of the cell wall, while the white arrows indicate the displaced plasma membrane due to plasmolysis.

Plasmolysis experiments were performed to distinguish whether the protein localizes to the apoplast or resides at the plasma membrane or within the cytoplasm (Figure 6). Free eGFP co-localizes with the cytoplasmic mCherry marker, confirming that both proteins are retained within the cytoplasm (Figure 6A). However, the two LOC_Os04g40510 localization constructs are found in distinct cellular compartments. The NtP-GFP-LOC_Os04g40510 resides within the apoplast and the cell wall, suggesting extracellular targeting of the fusion protein (Figure 6B). As the fluorescent signal was relatively weak for LOC_Os04g40510-GFP, the localization pattern during plasmolysis could not be clearly distinguished. The LOC_Os04g40510-GFP co-localizes partly with the free mCherry protein and near the cell nucleus (Figure 6C).

### 2.4. Characterization of Transgenic Lines

**Figure 7:**
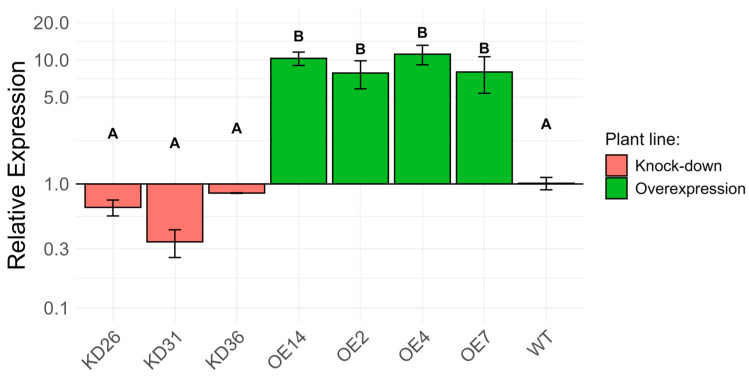
Transcript profiling of LOC_Os04g40510 in different transgenic lines of 1 DPI seed normalized to the transcript level in wild-type seedlings (log-scale). Plant material of 10 whole seedlings, grown on agar plates, were pooled to one biological replicate. For each time point, three biological replicates were used. Expression was analyzed using a one-way ANOVA followed by multiple hypothesis correction using the Tukey–Kramer method. The error bars represent the standard error of the samples and the letters on top of the error bars indicate the significance level between transgenic lines at the *p* < 0.05.

Transcript levels for *LOC_Os04g40510* in the different overexpression and knock-down lines were determined through RT-qPCR in 1 DPI-old seedlings (Figure 7). The relative expression levels for *LOC_Os04g40510* in the knock-down lines were different to those in the wild-type plants. However, statistical analysis did not provide evidence for a significant difference. After normalization of expression relative to the expression in wild-type plants, the knock-down lines expressed the gene of interest at a range of 0.34 ± 0.09 to 0.84 ± 0.004 (mean ± standard error). Overexpression lines generally showed elevated expression of the *LOC_Os04g40510* gene. The fold change in the overexpression lines ranged between 7.85 ± 2.00 and 11.14 ± 2.00 compared to wild-type plants.

#### 2.4.1. Early Developmental Traits of Transgenic Lines

The seed germination rate of the transgenic lines was evaluated at 3 DPI (Supplementary Figure S4). Statistical analyses were performed but no significant differences were detected.

Overexpression and knock-down lines were evaluated by measuring shoot length, root length, and root-to-shoot ratio after growth of seedlings for 14 DPI (Supplementary Figure S5) in a hydroponic system. In general, no clear trends were observed in shoot length for the transgenic lines compared with wild-type plants. Only a single knock-down line (KD31) showed a significant increase in shoot length. However, the overexpression lines displayed significant differences, with either increased or decreased shoot length depending on the line, indicating no consistent effect of overexpression on shoot growth. For root length, no clear changes were observed in the knock-down lines, except for KD36. In contrast, a consistent trend was evident among the overexpression lines, as most lines exhibited significantly shorter roots relative to wild-type plants, except for OE4. The root/shoot ratio revealed no significant changes in the knock-down lines, whereas overexpression of *LOC_Os04g40510* resulted in decreased root/shoot ratios compared to wild-type plants.

#### 2.4.2. Late Developmental and Reproductive Traits of Transgenic Lines

At 20 weeks after imbibition (WPI), the plants were senescing and the seeds were harvested. An initial screening (performed in 2023) indicated a potential effect of the aberrant protein expression on the development of the transgenic lines (Supplementary Data S1). The data presented here were obtained from a more in-depth study containing a higher number of transgenic lines (performed in 2024). Statistical analysis revealed a significant seasonal effect, preventing the merger of the two datasets, likely due to variation in environmental and climatological conditions between growth seasons. Nevertheless, the overall trends observed in 2024 were consistent with those from the screening in 2023, highlighting the reproducibility of the observed phenotypic effects across seasons.

**Figure 8:**
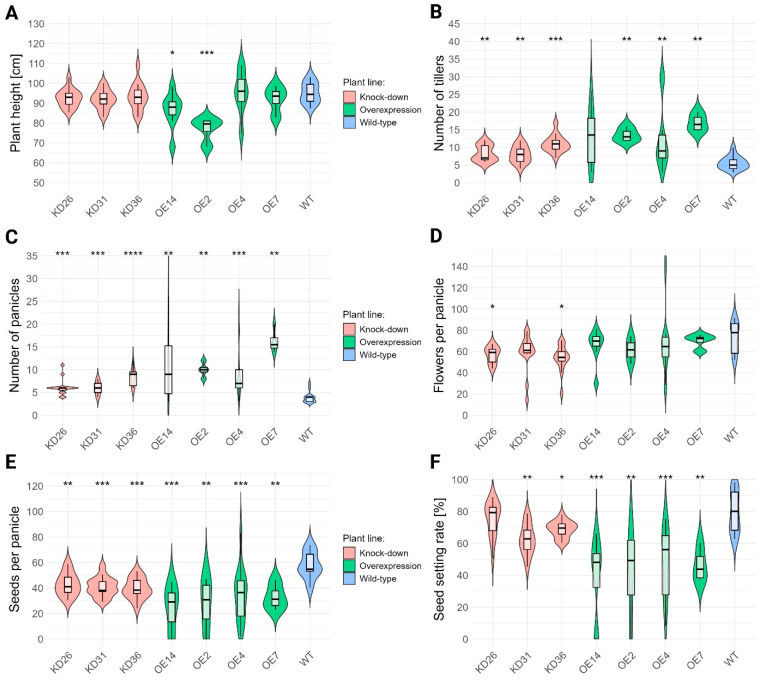
Vegetative and generative characteristics of transgenic lines. (**A**) Plant height was measured for senescent (20 WPI) T3 plants. (**B**) The tiller number was counted for the same plants. (**C**) Number of panicles per plant was counted for 20 WPI-old T3 plants. (**D**) The number of flowers per panicle was determined. (**E**) The number of seeds per panicle was counted for the same samples. (**F**) Based on the total number of seeds and the total number of flowers, the seed setting rate was calculated. Normality was evaluated through Shapiro–Wilk test and presence of homoscedasticity was determined by the Levene test. Non-parametric tests (Kruskal–Wallis followed by Wilcoxon rank sum test) were performed for all data given the absence of either normality and/or due to unequal variances. Multiple hypothesis correction was performed with Benjamini–Hochberg. The significant differences compared to the wild-type plants (WT) are denoted with ‘*’. The number of ‘*’ corresponds to the *p*-value: *p* < 0.0001: “****”, *p* < 0.001: “***”, *p* < 0.01 “**”, *p* < 0.05: “*”.

Little to no effect was observed for plant height in the knock-down lines (Figure 8A). Overexpression lines showed no consistent trend; two lines (OE2 and OE14) were significantly shorter than wild type, whereas the others did not differ significantly. In contrast, tiller number was strongly affected in the transgenic lines, except for OE14 (*p* = 0.0539) (Figure 8B). Both knock-down and overexpression lines produced more tillers than wild-type plants. Knock-down lines showed an approximate 48.8–96.4% increase, whereas overexpression lines showed a stronger effect of 126.8–203.6%. These results indicate a correlation between *LOC_Os04g40510* expression and rice tillering.

The development of shoot growth, panicle number, and tiller number was monitored over a period of three to four months (Supplementary Figure S6).

Changes in the expression of *LOC_Os04g40510* resulted in a significant increase in the number of panicles in all transgenic lines (Figure 8C). Knock-down lines generated approximately 55.0–117,1% more panicles, whereas overexpression lines produced 141.1–318.6% more panicles than wild-type plants. In overexpression lines, the number of flowers per panicle was not affected but reduced significantly by 23.0–26.8% in several knock-down lines (Figure 8D). In contrast, the number of seeds per panicle reduced significantly for all transgenic lines(Figure 8E). Knock-down lines showed a reduction of 26.0–31.7%, whereas overexpression lines displayed a much stronger reduction (40.7–57.6%) in number of seeds compared with wild-type plants.

The seed setting rate for a plant is defined as the ratio of the total seed number over the total number of flowers. The total number of flowers per plant was estimated by the sum of the total number of seeds per plant and the total number of empty husks per plant. Seed setting rate was reduced in nearly all transgenic lines (Figure 8F). Knock-down lines, except for KD26, showed a 13.8–21.7% decrease relative to wild-type plants. Overexpression lines revealed a stronger reduction of 38.3–50.4%. The shift in seed setting could be attributed to a lower seed number or a higher number of flowers due to the higher number of panicles per plant. Upon further examination of seed and flower counts per plant, the average number of seeds was seemingly unaffected. However, *p*-values associated with the knock-down lines were 0.0509. The average number of flowers in the transgenic lines was significantly different compared to the wild-type plants (Table 1). The knock-down lines had significantly more seeds and flowers per plant compared to the wild-type plants. In contrast, the overexpression lines did not display a significant increase or decrease in seed number per plant, while the lines OE14 and OE7 showed a significant increase in the number of flowers compared to the wild-type plants. Furthermore, the majority of the OE lines had lower seed counts with the exception of OE7.

#### 2.4.3. Seed Characteristics of Transgenic Lines

A comparative analysis of different seed traits was performed for the seeds from transgenic and wild-type seeds (Supplementary Figure S7). The mass of 50 seeds per plant was significantly reduced in several lines compared to wild-type plants, especially in the knock-down lines, except for KD31. Knock-down lines showed a significant 5% reduction in the mass of 50 seeds compared to wild-type seeds.

**Figure 9:**
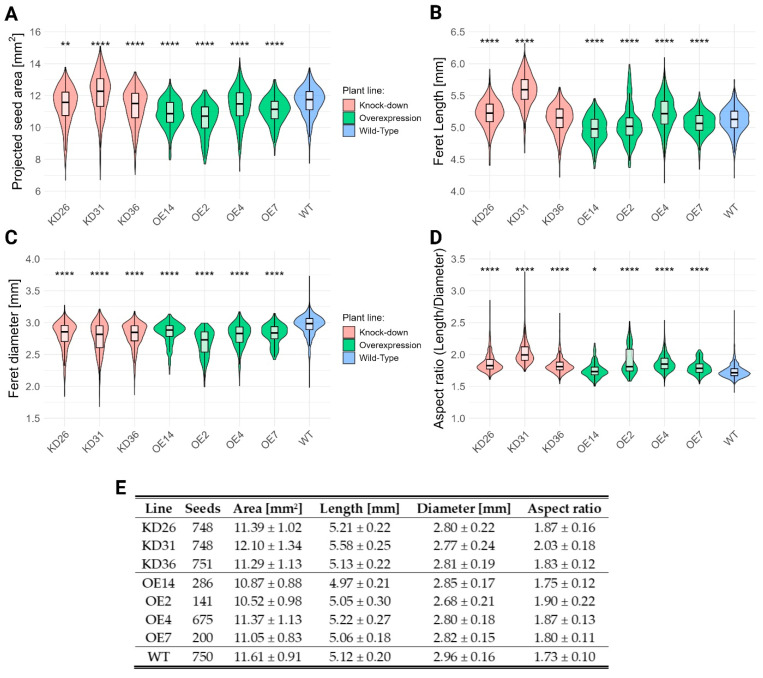
Seed morphology traits across transgenic lines. Panels (**A**–**D**) show the distribution of the tested morphological traits: (**A**) seed area, (**B**) length, (**C**) width, and (**D**) aspect ratio. Seed length was defined as the Feret diameter, representing the longest distance between two parallel tangents to the seed contour. Seed width was defined as the minimum Feret diameter. Panel (**E**) summarizes the number of seeds used for the analysis and the average values for each trait. Normality was evaluated through Shapiro–Wilk test and presence of homoscedasticity was determined by the Levene test. Parametric tests (pairwise *t*-test) were performed for all data, given the number of seeds analyzed, resulting in the central limit theorem. Multiple hypothesis correction was performed with Benjamini–Hochberg. The significant differences compared to the wild-type (WT) are denoted with ‘*’. The number of ‘*’ corresponds to the *p*-value: *p* < 0.0001:“****”, *p* < 0.01 “**”, *p* < 0.05:“*”.

Pictures of the same T4-seeds were analyzed for projected seed area, seed length, seed diameter, and aspect ratio for each seed (Figure 9). The transgenic seeds showed a reduced seed area compared to the seeds from wild-type plants, with the exception of the KD31 line, which had on average a 4.2% increase in seed area. The projected seed size reduction ranged from 1.9% to 2.8% in the knock-down lines and from 2.1% to 9.4% in the overexpression lines. The seed length differed from the transgenic lines compared to the wild-type plants. Generally, the seeds from the knock-down lines were longer, ranging from 0.2% to 9.0% increase. In the case of overexpression lines, seed length generally decreased by 1.2% to 2.9% compared to wild-type seeds, with the OE4-line being the exception (2.0% increase). In addition, seed diameter decreased for all transgenic lines, 5.1% to 6.4% for the knock-down lines and 3.7% to 9.5% for the overexpression lines. The seed aspect ratio for the transgenic seeds is generally higher compared to the wild-type seeds, which is the result of the reduction in seed diameter. The seed aspect ratio increased approximately with 5.8–17.3% for the knock-down lines compared to the wild-type seeds, and varied on average between 1.2% and 9.8% for the overexpression lines.

#### 2.4.4. Seed Chalkiness, Notched Belly, and Grain Surface Crease Analysis

**Figure 10:**
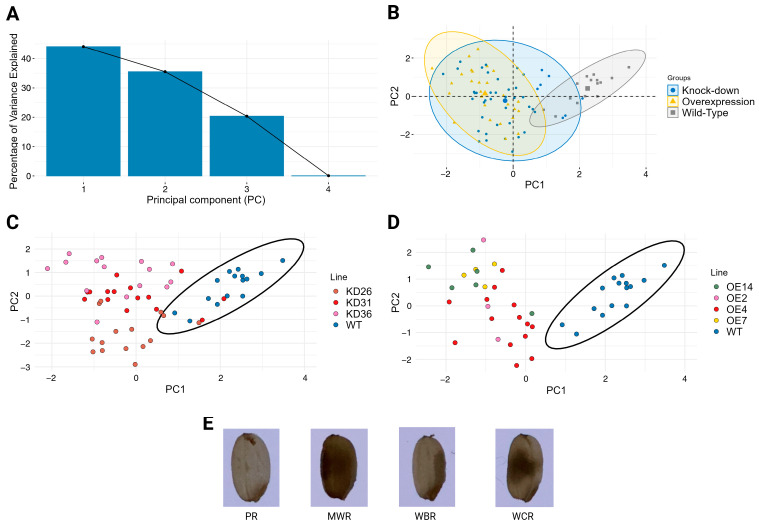
PCA of percentage seed chalkiness in different transgenic lines. (**A**) Shows the scree plot denoting the percentage of variance explained by each principal component (PC). (**B**) Displays the seed chalkiness distribution for the different transgenic lines focusing on the type of expression. PCA for the knock-down lines (**C**) and the overexpression lines (**D**) were compared with the wild-type seeds. (**E**) Images of the most dominant types of seed chalkiness: perfect rice (PR), milky-white rice (MWR), white-belly rice (WBR) and white-core rice (WCR).

Next to the seed size parameters, grain quality-related traits such as chalkiness, notched belly, and grain surface creases were assessed. To evaluate the variation in seed chalkiness, principal component analysis (PCA) (Figure 10) was performed based on the percentage of transgenic T4 seeds and wild-type controls displaying the distinct types of chalkiness. To enhance the PCA interpretability, the most frequent chalkiness phenotypes were retained for the analysis. These features were perfect rice (PR), white-belly rice (WBR), white-core rice (WCR), and milky-white rice (MWR). The first two principal components explain 79.59% of the total variation in chalkiness (PC1: 44.07% and PC2: 35.52%). PC1 represented the main axis of variation, distinguishing translucent seeds (PR, loading 0.69) from those with predominantly chalky phenotypes (WBR: −0.11, WCR: −0.52, and MWR: −0.48). PC2 was mainly defined by the WBR phenotype (loading −0.83), whereas the other phenotypes had mild to moderately positive contributions (PR: 0.30, WCR: 0.46, and MWR: 0.13).

**Figure 11:**
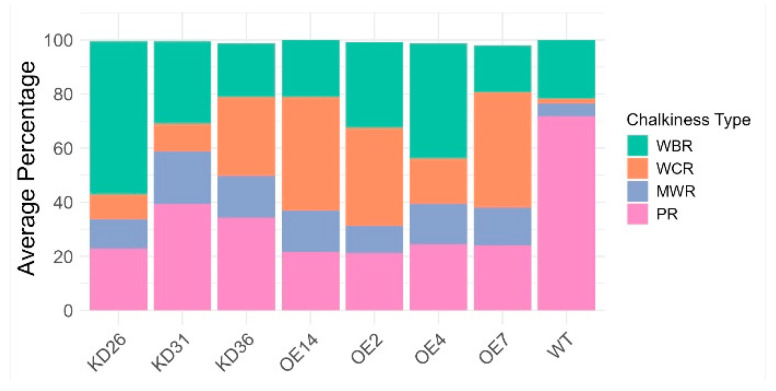
Stacked bar chart showing the average percentage composition of chalkiness types: perfect rice (PR), white-belly rice (WBR), white-core rice (WCR), and milky-white rice (MWR) across individual transgenic lines. Each bar represents the mean proportion of seeds exhibiting each chalkiness phenotype per line. Normality was evaluated through Shapiro–Wilk test and presence of homoscedasticity was determined by the Levene test. Non-parametric tests (Kruskal–Wallis followed by Wilcoxon rank sum test) were performed for all data given the absence of either normality and/or due to unequal variances. Multiple hypothesis correction was performed with Benjamini–Hochberg. Adjusted *p*-values can be found in Supplementary Data S2.

PCA based on the chalkiness percentage profiles revealed distinct expression-type dependent patterns. Seeds from overexpression lines and wild-type plants clustered largely separately, with only a slight overlap observed in their 95% confidence ellipses, indicating a shift in chalkiness traits due to gene overexpression. However, the knock-down lines exhibited a more intermediate or transitional phenotype with their distribution enveloping both the overexpression line and part of the wild-type clusters. The stacked bar chart (Figure 11) displays clear shifts within the distinct types of chalkiness at the level of transgenic lines. The proportion of fully translucent (non-chalky) rice was reduced in all transgenic lines relative to wild-type plants. Approximately 71.9% of the wild-type seeds were translucent. In the knock-down lines, 22.8–39.3% of seeds were non-chalky, whereas in the overexpression lines, 21.3–24.5% of the seeds did not display a chalky phenotype. Moreover, the overexpression lines generally show higher numbers of white-core rice compared to the knock-down lines and wild-type plants. The majority of the chalky seeds in the wild-type plants are from the white-belly type. Additionally, milky-white rice increased for both the overexpression lines and the knock-down lines. KD36 distinguishes itself from the other knock-down lines, given that KD36 showed on average a higher percentage of white-core rice compared to other knock-down lines. Similar trends for chalkiness types are observed between the overexpression lines, with the exception of OE4, which had a lower percentage of seeds of the white-core rice and a relatively higher percentage of white-belly rice. Overall, irrespective of line-specific variations, transgenic lines showed a clear increase in chalky seeds relative to wild-type plants. Statistical analysis indicated that the proportions of all chalkiness types differed significantly from those of the wild-type plants (*p* < 0.05), except for the white-belly type (WBR) in lines OE14, OE2, OE7, and KD36 (Supplementary Data S2).

**Figure 12:**
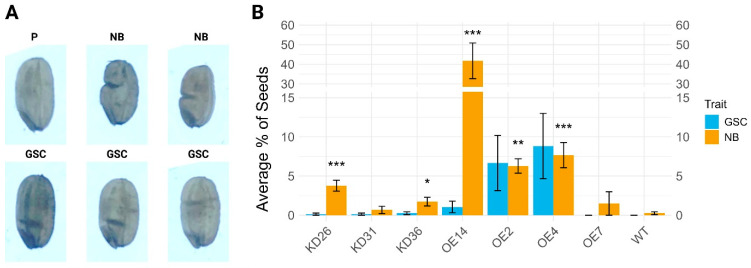
Data for notched belly and grain surface crease phenotypes for different transgenic lines. (**A**) Normal seed (P) and examples of seeds having a notched belly (NB) and surface crease (GSC). (**B**) Bar plot showing the average percentage of seeds with a notched belly (NB) and surface crease (GSC). Bars represent mean values ± standard error. Statistical significance for NB (relative to wild-type) was assessed using the Wilcoxon rank sum test with Benjamini–Hochberg correction. The significant differences compared to the wild-type (WT) are denoted with ‘*’. The number of ‘*’ corresponds to the *p*-value: *p* < 0.001: “***”, *p* < 0.01: “**”, *p* < 0.05: “*”). No significance testing was displayed for GSC.

Some very distinct kidney-shaped seeds, also known as the notched-belly (NB) grains, or seeds displaying a grain surface crease (GSC) were observed for some of the transgenic seeds (Figure 12). The presence of the notched belly (NB) and surface crease (GSC) traits in transgenic rice lines is different compared to the distribution in wild-type plants. Overall transgenic lines have more seeds with NB and/or GSC, with some exceptions for KD31 and OE7. The overexpression lines have higher occurrence of NB and GSC compared to the knock-down lines. The surface creases within seeds were practically absent in the knock-down lines and the wild-type plants. This is not the case for most of the overexpression lines, namely OE14, OE2, and OE4. Overexpression line OE7 and the wild-type plants did not have any seeds with surface creases.

## 3. Discussion

Sequence analyses revealed strong conservation within each subfamily but also highlighted clear differences between them. In addition, domain architecture also differed notably between subfamilies. With the exception of *LOC_Os01g47400*, rice GH5_7 enzymes are predicted to lack signal peptides, whereas GH5_11 and GH5_14 members generally possess a signal peptide. GH5_11 enzymes feature a *C*-terminal ricin B-like lectin domain, while GH5_14 enzymes contain a β-trefoil domain (annotated as DUF7910 or Fascin-like) following the first β-strand of the catalytic domain [23,24]. Whether these β-trefoil structures, ricin B-like or Fascin-like domain, function as auxiliary carbohydrate-binding modules remains unclear and requires further investigation [33].

To understand the expansion of the GH5 family in rice, gene duplication analysis was performed. Chromosome mapping revealed that GH5 genes are distributed across multiple chromosomes, with the GH5_7, GH5_14, and GH5_11 subfamilies showing progressively less dispersed patterns. This distribution was consistent with the observed duplication types: GH5_11 subfamily primarily expanded through short-range duplication events such as proximal and tandem duplications, whereas the GH5_7 subfamily mainly originated from dispersed duplications and DNA-transposed duplications.

The fate of duplicated genes can vary, leading to neofunctionalization (acquisition of a new function), subfunctionalization (partitioning of the ancestral function), non-functionalization (pseudogene formation), … [37,38]. In some GH5 genes (*LOC_Os01g47400_2*, *LOC_Os03g61270*, and *LOC_Os11g02600*), incomplete TIM-barrel domains were observed, or key catalytic residues were missing (*LOC_Os01g47400_2* and *LOC_Os11g02600*). In contrast, other genes (*LOC_Os01g54300, LOC_Os10g22570*, and several GH5_11 enzymes) contained additional domains, suggesting potential acquisition of new functions. Collectively, these structural variations may indicate the emergence of novel or modified functions, or in some cases, the loss of enzymatic activity. The possibility that some atypical GH5 sequences correspond to misannotated or partial gene models cannot be excluded, as such annotation errors have been reported in previous rice genome releases [39,40].

Sequence analysis predicted that *LOC_Os04g40510* encodes a protein with an *N*-terminal signal peptide, suggesting targeting to the secretory pathway and possible secretion to the apoplast or plasma membrane [41,42]. To experimentally validate the prediction, subcellular localization experiments were performed. Microscopy images for two distinct localization constructs, namely LOC_Os04g40510-GFP and NtP-GFP-LOC_Os04g40510, suggest that the GFP-tagged proteins are targeted to different cell compartments. The vesicle-like structures and nuclear periphery, observed for the *C*-terminally tagged protein, could correspond to ER- and/or Golgi-related vesicles [43,44], although localization to other secretory compartments, for example extracellular vesicles, cannot be excluded. Unfortunately, fluorescence from LOC_Os04g40510-GFP was weak at the cell periphery, and signal intensity after plasmolysis was insufficient to confidently assign localization to the apoplast. However, NtP-GFP-LOC_Os04g40510 is clearly secreted to the apoplast. The subcellular localization experiments demonstrate that the protein is found in the secretory pathway. These results should be interpreted with caution, as GFP tagging and protein processing can influence localization [45,46,47,48]. The vesicle-like pattern observed for the *C*-terminal fusion is consistent with ER/Golgi-associated trafficking, whereas the apoplastic signal of the internal GFP fusion supports a role in secretion. Together, the data are consistent with LOC_Os04g40510 functioning in the secretory pathway.

Phenotyping experiments with overexpression lines and knock-down lines revealed diverse effects of the aberrant expression of *LOC_Os04g40510* compared to wild-type plants. At 3 DPI, no significant differences were observed for the germination rates between transgenic and wild-type plants. However, at 14 DPI, root length of the overexpression lines was significantly reduced. Altered cell wall composition is known to affect root growth and stress tolerance [49,50,51,52,53,54]. For instance, knock-out mutants of Golgi-localized exo-β-1,3-galactosidases in *Arabidopsis thaliana* displayed reduced root growth [49], whereas absence of the *A. thaliana* β–glucuronosyltransferase *AtGlcAT14A* in knock-out mutants enhanced root cell elongation [52]. These observations highlight how abnormal cell wall dynamics can influence root development, consistent with the shortened roots observed in *LOC_Os04g40510* overexpression lines in this study.

Tiller numbers per plant were significantly increased for both the overexpression lines (141.1–318.6%) and the knock-down lines (55.0–117.1%). A similar link between cell wall-associated enzyme activity and tiller number has been reported in wheat. Specifically, near-isogenic lines (NILs) carrying the *tiller inhibition* (tin) allele, which is linked to a cellulose synthase-like (*Csl*) gene, exhibited thicker, lignified cell walls, fewer tillers, and increased grain weight compared to the free-tillering cultivar [55]. In rice, *LOC_Os04g40510* may influence tillering through related changes in cell wall properties. The moderate increase in tiller number in knock-down lines improved overall yield, despite producing smaller seeds with reduced grain weight. Overexpression lines generated at least twice as many tillers per plant as wild-type plants. The increase in tillering for both transgenic types presumably affected seed setting due to an increase in flower number.

Although tiller number indirectly determines rice yield by influencing the number of productive panicles [56], higher tiller counts do not always translate to increased seed setting, as shown in the overexpression lines. Excessive tillering or late emerging tillers often reduce yield and, in the latter case, cause unproductive panicles [56,57]. Wang et al. (2017) and Kalaitzidis et al. (2025) also report that late-emerging tillers often contribute little to final grain yield and may suffer higher rates of abortion or poor seed setting [58,59]. Time-course analysis showed that overexpression lines developed more late-emerging tillers and panicles, which was likely associated with yield loss. Knock-down lines also produced more tillers, though to a lesser extent, relative to overexpression lines, and tillering and panicle development was almost completely arrested by 12 WPI, similar to wild-type plants. These results indicate that altered *LOC_Os04g40510* expression is correlated with changes in the extent and timing of tiller emergence, which may contribute indirectly to the observed effects on seed yield. However, the underlying mechanism remains to be clarified.

Whether the fertility traits are solely the result of tillering adversities still requires further analysis. However, additional molecular mechanisms may influence fertility. Knock-down lines and overexpression lines exhibited a reduced seed setting rate due to increased flower numbers which mainly resulted in empty husks. A reduction in seed setting was also reported for the *LOC_Os04g40510* paralog, *LSSR1*, where knock-out mutants exhibited a reduced seed setting rate, which was attributed to abnormal pollen grain germination, failed pollen tube penetration, and retarded pollen tube elongation [17]. Perturbation of the rice GH5_11 expression, namely *LSSR1* and *LOC_Os04g40510*, influences fertility, though the underlying mechanisms are likely distinct given their divergent spatiotemporal expression patterns.

Expression analysis confirms that LOC_Os04g40510 is localized within the rice seed, with promoter activity in the endosperm. Database mining showed that *LOC_Os04g40510* was highly expressed around 10 days after pollination (DAP). At this developmental stage, the embryo is near to completing the morphogenetic differentiation and focuses on its organ enlargement and maturation [60]. The endosperm starts to accumulate significant amounts of starch and storage proteins, also known as the grain filling stage [61,62]. This spatiotemporal pattern aligns with the phenotypic observations of altered seed traits, such as chalkiness and notched belly, pointing to a role for *LOC_Os04g40510* in seed development.

Notched belly can already be observed 5 days after anthesis [20] but can still emerge at later stages [63]. The occurrence of white core and white belly chalky seeds is caused during early to later stages of grain filling [23]. Several seeds from the transgenic lines show chalkiness within the endosperm, notched belly, and grain surface creases. PCA of chalkiness highlights that transgenic lines contain seeds with enriched rice chalkiness. However, knock-down lines show transitional chalkiness characteristics between wild-type plants and overexpression lines. Notched belly and grain surface crease were most pronounced in overexpression lines, although knock-down lines also displayed slightly higher levels of these traits compared to wild-type seeds. The grain surface crease phenotype observed in this study has been rarely reported in rice, suggesting it may be an overlooked developmental irregularity. The phenotype is possibly caused by similar processes underlying the notched belly phenotype but occurring on the surface rather than on the ventral side (belly) of the rice grain. The occurrence of the notched belly phenotype can be caused by a range of factors, such as environmental and genetic cues. Importantly, both chalkiness and notched belly negatively affect milling quality and market value, highlighting their agronomic relevance.

Notched belly and chalkiness are created at the grain filling stage and are often the result of improper source-sink partitioning [63,64,65]. A study examining the embryo–endosperm interface reported that the endosperm was metabolically converted into a nutrient source for the developing embryo, leading to impaired storage compound accumulation and the formation of a chalky phenotype [65]. In a similar study, seeds with notched belly and (non)-chalky phenotype were used to indicate the crosstalk between embryo and endosperm [66]. While *LOC_Os04g40510* expression and phenotypic effects suggest a possible involvement in these processes, our current data do not directly demonstrate an effect on source–sink partitioning. Future biochemical and metabolic analyses will be required to clarify whether *LOC_Os04g40510* influences grain filling directly through endosperm or cell wall modification, or indirectly through altered sugar partitioning. Several phenotypic changes, including increased chalkiness, reduced seed-setting rate, and a higher number of tillers and panicles, were observed in both the overexpression and knock-down lines compared with wild-type plants, although the effects were generally more pronounced in the overexpression lines. Given the sequence similarity among GH5_11 members, functional redundancy or compensatory activity by related enzymes may mask the impact of reduced *LOC_Os04g40510* expression. Alternatively, metabolic buffering mechanisms could compensate for altered expression levels, or the relationship between *LOC_Os04g40510* expression and phenotype may be non-linear, with either elevated or reduced activity disturbing normal development. Further analyses at the transcriptional and metabolic levels will be required to evaluate these hypotheses.

The study of the *LOC_Os04g40510* gene revealed that the protein of interest appears to be trafficked via the classical secretory pathway and is predominantly localized in the seed endosperm. Altered expression of *LOC_Os04g40510* affected multiple phenotypic and morphological traits in rice, including increased occurrence of chalky seeds, notched belly grains, and grain surface creases. In parallel, tillering and panicle development were enhanced in transgenic lines. Reduced seed setting rate was also observed in overexpression and knock-down lines but in the case of the knock-down lines improved the overall seed number per plant. These findings highlight the importance of cell wall active enzymes in rice development, specifically in tiller number, fertility, and endosperm development. Although the precise biochemical activity of the LOC_Os04g40510 still needs to be determined, its impact on agronomic traits suggests that it may be a promising target for breeding strategies aimed at optimizing rice yield and grain quality. However, comprehensive follow-up studies, including transcriptomic and metabolic analyses, will be essential to validate its functional role and assess its potential for crop improvement.

## 4. Materials and Methods

### 4.1. Database Mining and Sequence Analysis

The GH5 sequences in rice were extracted from the Phytozome online database [67] by using the ‘IPR001547’ InterPro identifier and the ‘*Oryza sativa v7.0*’ genome [68], which corresponds to the *Oryza sativa* spp. japonica cv. Nipponbare. A total of 16 distinct genes were identified as part of the InterPro cluster (Supplementary Data S3: Sequences). During the search, several genes (*n* = 4) were found to have multiple splicing variants. However, upon further inspection employing ClustalOmega (https://www.ebi.ac.uk/jdispatcher/msa/clustalo, accessed on 25 August 2025) [69], only two genes displayed splicing variants for which the amino acid sequence was different, namely LOC_Os01g47400 and LOC_Os03g61280. The genes were mapped onto the rice genome with the help of ePlant rice [70] and PLAZA dicots v5.0 [71].

The duplication events were assessed using the *doubletrouble* database (doubletroub https://almeidasilvaf.github.io/doubletroubledb/, accessed on 30 August 2025), namely the ‘IRGSP-1.0’-assembly [72]. Using this tool, additional genes were found, namely *LOC_Os03g61270* and *Os04g0276300*; the former was kept for analysis whilst the latter was omitted from the analysis. LOC_Os03g61270 was predicted by CUPP [73] to be part of the GH5 family, while Os04g0276300 was not classified, presumably due to being too short (98 amino acids). The gene pair clusters resulting from the *doubletrouble* database were visualized using Cytoscape v. 3.10.2 [74] (Supplementary Data S3: Duplication).

GH5 sequences were submitted to several online tools to evaluate distinctive characteristics. The presence of a putative signal peptide was investigated by SignalP v6.0 [75,76] (Supplementary Data S2: Signal Peptide). The following parameters were used: ‘Eukarya’, ‘Long output’, and the ‘Fast’ model mode was selected. The domain architecture of the enzyme was evaluated using the InterPro server [77] (Supplementary Data S3: Domain Architecture). Domain and GH domains of the enzymes were classified by CUPP into their putative subfamilies [73].

Protein structures were obtained from the AlphaFold protein structure database [78,79].

Amino acid sequences of the GH5 domains were aligned using MAFFT v7 FFT-NS-2 [80]. The best-fit model for amino acid substitution was determined using ModelTest-NG v0.1.7 [81] under Bayesian Information Criterion (BIC) (seed = 12345, threads =4). The WAG+I+G4 model was identified as optimal for the selected GH5 sequences. Subsequently, IQ-TREE 2 v. 2.3.6 [82] was employed to construct phylogenetic trees based on maximum likelihood. Branch support was evaluated using 1000 ultrafast bootstrap replicates (UFBoot2) [83] and 1000 SH-aLRT replicates (seed = 12345, threads = 8, Intel Xeon Gold 6240 (Cascade Lake @ 2.6 GHz) processor from the high performance computing (HPC) infrastructure VSC (Flemish Supercomputer Center), Ghent, Belgium). The trees were visualized in Rstudio v. 4.4.2. The same multiple sequence alignment was analyzed and visualized with ESPript v. 3.0 (https://espript.ibcp.fr/ESPript/cgi-bin/ESPript.cgi, accessed on 15 September 2025) [84,85]. Structural images were visualized with Pymol v. 2.5.2 [86].

Publicly available RNA seq data from the Expression Atlas database [87,88,89,90,91] enabled us to examine tissue-specific and developmental expression patterns of LOC_Os04g40510 (accessed on 23 February 2025). Reported values represent normalized expression levels in transcripts per million (TPM), pre-averaged from the technical and biological replicates available within each dataset. Multiple datasets from independent studies were available for seed-related samples, allowing calculation of mean TPM values.

### 4.2. Plant Materials

Seeds of *Nicotiana benthamiana* were germinated in soil hydrated with 1 g/L fertilizer (Soluplant 19-8-16+4MgO+ME (Intergrow, Aalter, Belgium)) in a growth chamber at 24.5 °C with a light regime of 16 h light/8 h dark and a relative humidity of 75%. The light intensity was 150 µmol/m^2^s. After 2 weeks, the seedlings were transferred to individual pots for further growth. Plants were watered every three days with water or fertilizer (1 g/L Soluplant 19-8-16+4MgO+ME (Intergrow)) in an alternating pattern.

*Oryza sativa* L. subsp. japonica cv. Kitaake seeds were used throughout the distinct experiments. Transgenic lines were generated using *Agrobacterium tumefaciens (strain EHA105)*-mediated transformation and were obtained from Biogle GeneTech (Changzhou, China). The knock-down (KD) lines were created by expressing an RNA interference (RNAi) cassette consisting of a 280 bp fragment of the 5′ coding region of LOC_Os04g40510 arranged in an inverted repeat (hairpin) configuration (Supplementary Data S3: RNAi cassette). Both the RNAi cassette (for KD lines) and the coding sequence of LOC_Os04g40510 (for OE lines) were cloned into the binary vector pYQ202 using restriction enzymes KpnI/BamHI for the KD construct and KpnI/SacI for the KD construct. Expression of these constructs was driven by the constitutive maize ubiquitin promoter and the nopaline synthase (NOS) terminator. In addition, the vector contains a hygromycin resistance gene for plant selection.

For promoter analysis, the native promoter sequence of the LOC_Os04g40510 (2428 bp fragment) was cloned upstream of the β-glucuronidase (GUS) gene into pCAMBIA1301 using the restriction sites NcoI/KpnI. The GUS gene was followed by the NOS terminator. The vector also contained the hygromycin resistance marker gene.

### 4.3. Sterilization and Germination of Rice

De-husked rice seeds were immersed in 70% ethanol (AnalytiChem, Zedelgem, Belgium) for 5 min while shaking. The ethanol was replaced by 5% (v/v) NaOCl for 30 min while shaking gently. The seeds were washed with autoclaved water at least 6 times and incubated overnight on a rotary shaker at 28 °C.

Seeds were germinated on Murashige and Skoog (MS) agar plates (4.3 g/L MS with modified vitamins (Duchefa, Haarlem, The Netherlands), 15 g/L sucrose and 12 g/L plant agar, pH 5.7) without or with 35 mg/mL hygromycin for either non-selective or selective medium. Transgenic seeds were grown on selective medium while wild-type seeds were grown on non-selective medium, unless stated otherwise. The medium was supplemented with 0.2 mg/mL Thiram 80WG (dimethylcarbamothioylsulfanyl-N,N-dimethyldithiocarbamaat, Eastman, Kingsport, TN, USA) to prevent fungal growth. The plates were kept in a growth chamber at 28 °C with a photoperiod (150 µmol/m^2^/s) of 12 h light/12 h dark, and a humidity of approximately 75%.

For the phenotyping experiment and seed multiplication (T2 => T3), 2-week-old seedlings from either the selective or non-selective plates were transferred to pots of hydrated soil (3 L) and grown in the greenhouse (26–28 °C) (UGent, Melle, Belgium). Young seedlings were watered twice weekly with approximately 100 mL of 6.5 mM FeSO_4_ and 6.8 mM (NH_4_)_2_SO_4_ for 6 weeks after transferring to soil.

### 4.4. Seed Multiplication and Characterization of Transgenic Rice Plants

Wild-type and transgenic plants were cultivated as described above. Approximately 5 weeks after imbibition, a 3–4 cm leaf tip was sampled from each T2-plant and placed in a sterile Safe-Lock tube. Plant material was homogenized using a TissueLyser II (Qiagen, Venlo, The Netherlands) with cooled adapters and three stainless steel beads (3 mm Ø) per sample for 30 s at 30 Hz. DNA extraction was performed using 1 mL of DNA extraction buffer (2% (w/v) CTAB (Sigma-Aldrich, Diegem, Belgium), 0.1 M Tris-HCl pH 7.5, 1.4 M NaCl, 2 mM EDTA) for 0.1 g of plant material. After chloroform: isoamyl alcohol (24:1) extraction DNA was precipitated with 100% isopropanol. The resulting DNA pellet was washed first with 76% (v/v) ethanol/0.2 M NaOCl followed by washing with 76% (v/v) ethanol/10 mM NH_4_OAc. DNA was dissolved in 50 µL of autoclaved distilled water and stored at −20 °C. The presence of the construct was confirmed through PCR by amplifying the hygromycin resistance gene. Amplification of the hygromycin resistance gene was achieved using 2 µL of DNA solution as template with primers A479/A480 and Taq DNA polymerase (VWR, Oud-Heverlee, Belgium). The PCR program consisted of an initial denaturation of 5 min at 95 °C followed by 35× (30″-95 °C, 30″-52 °C, 30″-72 °C) and a final elongation at 72 °C for 5 min.

### 4.5. Expression Analysis

Transcript levels for LOC_Os04g40510 were quantified in seedlings grown in vitro during different germination stages and at 1 DPI for the distinct transgenic lines. A total of 10 seedlings were sampled and pooled to represent 1 biological replicate. Samples were frozen in liquid nitrogen and ground to a fine powder using a pre-chilled mortar and pestle.

Total RNA was extracted from the different tissues using the Spectrum Plant Total RNA—kit (Sigma-Aldrich). RNA was precipitated by adding 25 µL of 8 M LiCl to 50 µL of the RNA sample. The mixture was vortexed for 30 s and incubated overnight at −20 °C. Afterwards, the samples were centrifuged for 20 min at 15,000 rpm and room temperature. The RNA pellet was washed using 500 µL 75% (v/v) ethanol and centrifuged for 5 min at 7500 rpm. The pellet was resuspended in 30 µL of distilled water. The DNase I—kit (Thermo Fisher Scientific, Waltham, MA, USA) was used to remove residual DNA fragments. In short, 16 µL RNA sample was mixed with 2 µL DNAse buffer (10×) and 2 µL of DNAse I. The solution was vortexed for 10 s and incubated at 37 °C for 30 min. Next, 1 µL of 50mM EDTA was added to the solution and incubated for 10 min at 65 °C. cDNA synthesis was performed using the Maxima kit (Thermo Scientific). In short, 0.5 µg of DNAse-treated RNA was combined with 2 µL Maxima Enzyme Mix and 4 µL 5× Reaction Mix. The solution was incubated at 25 °C for 10 min followed by incubation at 55 °C for 20 min and 85 °C for 5 min. Subsequently, the cDNA sample was diluted 5 times using distilled water. Quality and quantity of the RNA and cDNA were determined using a NanoDrop2000 spectrophotometer (Thermo Fisher Scientific). In addition, the quality of the cDNA samples was analyzed through PCR using cDNA as a template. Therefore, 2 µL of the diluted cDNA, Taq DNA polymerase (VWR), and the primer set evd910/evd911 were employed. The PCR consisted of an initial denaturation of 5 min at 95 °C and 40× (30″-95 °C, 30″-58 °C, 30″-72 °C) and a final elongation step at 72 °C for 5 min.

RT-qPCR analysis was performed for the transcripts of interest and the reference genes (Supplementary Data S3: Primers). The mastermix for 1 reaction consisted of 1 µL for both the forward and reverse primer, 10 µL of iQTM SYBR Green Supermix, 10 µg of cDNA, and 6 µL of water. The RT-qPCR program was 3 min-95 °C and 41× (15″-95 °C, 25″-60 °C, 20″-72 °C). At least three biological replicates (with the exception of the OE2 line due to seed limitations) were analyzed and each biological replicate was evaluated in triplicate (three technical replicates).

### 4.6. Phenotypic Analysis of Rice Plants

Phenotypic analysis of transgenic rice was performed 3 days after imbibition (DPI), 14 DPI and 20 weeks after imbibition (WPI).

At 3 DPI, the germination rate was determined on in vitro grown seedlings. All seeds were sown on non-selective MS agar plates and grown in the plant growth room at 28 °C with a photoperiod (150 µmol/m^2^/s) of 12 h light/12 h dark and a humidity of approximately 75%. Four biological replicates were performed using at least 10 T3 seeds. The number of plants per line varied in the experiment (Supplementary Data S4: Phenotyping 3 DPI) for the different transgenic lines. In addition, the wild-type seeds with different chalkiness types: perfect rice (*n* = 31), white-core rice (*n* = 16), and white-belly rice (*n* = 13).

For the 14 DPI analysis, transgenic and wild-type seedlings were transferred to a hydroponic system at 6 DPI and grown in ½ Hoagland solution (2.5 mM KNO_3_, 0.5 mM KH_2_PO_4_, 2.5 mM Ca(NO_3_)_2_.4H_2_O 1 mM MgSO_4_.7H_2_O, 14 µM H_3_BO_3_, 4 µM MnSO_4_.4H_2_O, 0.15µM ZnSO_4_.7H_2_O, 0.015 µM (NH_4_)_6_Mo_7_O_24_.4H_2_O, 0.16 µM CuSO_4_.5H_2_O, 25 µM FeSO_4_.7H_2_O, 25 µM Na_2_EDTA.2H_2_O, pH 5.8) in the plant growth room. At 14 DPI, the shoot and root lengths were measured for at least four plants per biological replicate. The experiment was repeated three times (Supplementary Data S4: Phenotyping 14 DPI).

Phenotypic evaluation was conducted on transgenic plants (20 WPI) grown from T3 seeds (Supplementary Data S4: Phenotyping 20 WPI). The seeds were sown on MS agar plates and grown in the plant growth room. At 14 DPI, the plants were transferred to soil and were cultivated in the greenhouse. Shoot length, number of panicles, number of tillers, seed setting, and 50 seed weight were determined. Additionally, seed characteristics, seed morphology, and seed chalkiness were assessed for the T4 seeds obtained from the T3 plants. Moreover, images of approximately 50 seeds were randomly selected for each seed-bearing plant to determine the individual seed characteristics such as the mass, projected seed area, seed length, seed diameter, and length-to-diameter ratio. The number of seeds evaluated varied for each line (Figure 9E). FIJI was employed to analyze these images. The chalkiness was visually evaluated using transillumination and the chalky endosperm was classified based on the article of Yoshioka et al. (2007) [92]. The presence of the ‘Grain surface crease’ and the notched belly phenotypes were also assessed.

### 4.7. GUS Histochemical Staining Assay

Activity of the LOC_Os04g40510 promoter (2428 bp) was evaluated using GUS histochemical staining. Plant tissues collected for different developmental stages were placed in 90% (v/v) ice-cold acetone for maximum 30 min at 4 °C while shaking. Afterwards, the tissues were washed three times with 0.1 M phosphate buffer (28 mM NaH_2_PO_4_.2H_2_O and 72 mM NA_2_HPO_4_, pH 7.2). Each wash step was performed for 5 min while shaking at 21 °C. Subsequently, the tissue was incubated in GUS preincubation buffer (0.1 M phosphate buffer, 0.5 mM K-ferricyanide, and 0.5 mM K-ferrocyanide) for 10 min under vacuum conditions at 21 °C followed by incubation for 20 min at 37 °C. Next, the buffer was replaced by GUS preincubation buffer containing 2 mM X-glucuronide (Thermo Scientific), the tissues were kept under vacuum at 21 °C for 20 min and incubated overnight at 37 °C. The stained tissues were washed three times in 0.1 M phosphate buffer for 20 min while shaking and were stored in 70% (v/v) ethanol.

### 4.8. Subcellular Localization Experiments

The coding sequence of LOC_Os04g40510 was synthesized by GeneArt (Thermo Fisher Scientific, Waltham, MA, USA). Two distinct localization constructs for LOC_Os04g40510 were cloned: a *C*-terminal GFP fusion (LOC_Os04g40510-GFP) and a GFP insertion construct in which GFP is localized after the signal peptide (NtP-GFP-LOC_Os04g40510).

For the C-terminal GFP fusion (LOC_Os04g40510–GFP), the native stop codon was removed and *attB* recombination sites were introduced by two successive PCRs using primer pairs L555–L556 and evd002–evd004. The resulting fragment was cloned into pDONR221 via a BP reaction and subsequently transferred into the pK7FWG2 destination vector (containing a GFP tag in the backbone) by LR recombination (Gateway™, Invitrogen, Carlsbad, CA, USA).

For the *N*-terminal GFP fusion (NtP–GFP–LOC_Os04g40510), three fragments corresponding to (1) the predicted signal peptide, (2) the CDS lacking the signal peptide, and (3) GFP were amplified with primer pairs A193–A194, A195–A196, and evd95-evd96, respectively. The synthesized LOC_Os04g40510 CDS served as the template for the first two PCRs, while the empty vector pK7FWG2 was used as a template to amplify GFP. The three gel-extracted PCR fragments (QIAquick^®^ Gel Extraction Kit, Qiagen) were assembled by overlap-extension PCR (Q5 High-Fidelity polymerase, NEB). Each overlap-extension reaction consisted of 5 µL 5× Q5 buffer, 2 µL 10 mM dNTPs, 5 µL GC enhancer, 0.5 µL Q5 DNA polymerase, and an equimolar mixture of the three fragmented adjusted to a final volume of 25 µL. The thermal cycling conditions were 30″-98 °C, 15× (10″-98 °C, 90″-72 °C), 10′-72 °C, ∞-12 °C. The overlap-extension product generated the full-length construct containing half of the *attB* sites. Using primers evd002–evd004 and 1:5 dilution of the overlap-extension reaction as template, a PCR was performed to obtain the full-length product with complete *attB* sites. The resulting fragment was cloned into pDONR221 and recombined into the pK7WG2.0 destination vector (lacking a fluorescent tag in the backbone).

Intermediate PCR products amplified with primer set evd002-004 were verified by sequencing after blunt-end cloning into pJET1.2 (Thermo Fisher Scientific). All BP and LR recombination reactions were performed according to the manufacturer’s instructions (Gateway™ BP/LR Clonase™ II kits, Invitrogen). Final destination constructs were confirmed by Sanger sequencing (LGC Genomics, Berlin, Germany). Plasmids were propagated in *E. coli* TOP10 under the appropriate antibiotic selection and purified using the GeneJet plasmid miniprep kit (Thermo Fisher Scientific). Primers required to generate distinct subcellular localization constructs can be found in Supplementary Data S3 (Primers).

Destination vectors were transformed into electrocompetent *Agrobacterium tumefaciens* strain C58C1 pMP90 Rif^R^ by electroporation. The cells were selected on YEB agar medium (5 g/L beef extract (Lab M Ltd., Lancashire, UK), 5 g/L peptone (Merck, Darmstadt, Germany), 1 g/L yeast extract (Duchefa), 5 g/L sucrose, and 15 g/L agar) containing 20 mg/L gentamycin, 200 mg/L rifampicin, and 50 mg/L spectinomycin.

Sequences encoding free eGFP or free mCherry inserted in the pK7WG2 backbone, were used as a control. These constructs were already available in *A. tumefaciens* strain C58C1 pMP90 Rif^R^. Additionally, an *A. tumefaciens* strain expressing the tomato bushy stunt virus (TBSV) P19 protein was used to suppress post-transcriptional gene silencing (PTGS). The P19-expressing strain was grown in liquid LB medium supplemented with 50 mg/L kanamycin. The vectors pK7WG2.0, pK7FWG2, and pK7WGF2 were obtained from Plant Systems Biology (Vlaams Instituut voor Biotechnologie (VIB), Ghent, Belgium).

Four-week-old *N. benthamiana* leaves were co-infiltrated with *A. tumefaciens* strain C58C1 pMP90 Rif^R^ carrying the eGFP control vector, LOC_Os04g40510-GFP or NtP-GFP-LOC_Os04g40510, in combination with strains harboring the P19 silencing suppressor vector and the mCherry nucleocytoplasmic marker vector using a 1:1:1 ratio of *A. tumefaciens* suspensions (OD_600nm_ = 1) without wounding. Part of the infiltrated spot was excised from the tobacco leaf and mounted on a slide together with H_2_O to keep the sample moisturized. The slide was covered with a coverslip and sealed with nail polish. The Nikon A1R confocal microscope (Nikon, Tokyo, Japan) was used with a 40 × S Plan Fluor ELWD air objective lens (NA 0.60). The sample was excited at a wavelength of 561 nm using a diode laser. A dichroic mirror with 405/488/561 nm was selected. The emission filters were 500-550 and 553-618 for GFP and mCherry, respectively. The pinhole was set to 1 airy unit (AU). The unidirectional scanning was performed using a Galvano scanner with a 4× line averaging, a scan speed of 0.060 frames per second and a pixel size of 0.03 µm. Z-stacks of different samples were made. Image analysis was performed using FIJI and NIS-Elements Viewer version 5.21. To perform the plasmolysis, the water in the mounted samples was replaced with 5 M NaCl for maximum 2 min prior to imaging.

### 4.9. Data Analysis and Statistics

Data analysis of the qPCR experiment was performed with the Bio-Rad CFX Maestro and qBase+ software v. 3.2 [93]. The Bio-Rad CFX Maestro software v. 2.3 was employed to evaluate the melt curves for different samples. Preliminary analyses such as determination of primer amplification efficiency and stability were determined using the GeNorm algorithm in qBase+ [94,95]. Quality control of the technical replicates and selection of stable reference genes were also performed. The selected reference genes for the RT-qPCR of the germination stages were EXP (LOC_Os03g27010, evd910-evd911) and Fb15 (LOC_Os02g07910, A269-A270), while the reference genes EXP (evd910-evd911) and SAP18 (LOC_Os02g02960, A267-A268) were used for evaluating the expression in the transgenic lines at 1 DPI. The gene of interest, LOC_Os04g40510, was amplified with primers A276-A277. Statistical analysis was performed with qBase+ software.

A two-sided one-way ANOVA was performed to elucidate the significance for both RT-qPCR experiments, namely the expression analysis during the developmental stages during germination and the evaluation of the transcript levels in the transgenic lines. For the latter, the data was normalized to wild-type samples to see the degree of overexpression. Multiple hypothesis corrections were performed using the Tukey–Kramer method.

Normality and homoscedasticity of the phenotyping data from plants and seeds were evaluated by the Shapiro–Wilk test and by the Levene test, respectively. If the assumptions of normality and homoscedasticity were met, data were analyzed using one-way ANOVA followed by pairwise t-tests comparing each transgenic line to the wild-type plants. In cases where normality and/or homoscedasticity were violated, a non-parametric Kruskal–Wallis test was applied, followed by pairwise Wilcoxon rank sum test, comparing the transgenic lines with the wild-type plants. Multiple hypothesis corrections were performed using Benjamini–Hochberg procedure.

PCA of the chalkiness levels for the distinct transgenic lines was performed using RStudio. To enhance PCA interpretability and reduce noise, features occurring in less than 90% of samples were excluded.

## Figures and Tables

**Table 1:** Summary of average number of seeds and flowers per transgenic plant compared to wild-type plants. Normality was evaluated through Shapiro–Wilk test and the presence of homoscedasticity was determined by the Levene test. Depending on the results of the aforementioned analyses, parametric (ANOVA followed by *t*-test) or non-parametric tests (Kruskal–Wallis followed by Wilcoxon rank sum test) were performed. Multiple hypothesis correction was performed with Benjamini–Hochberg.

Line	Total Seed (Average)	*p*-Value	Total Flower (Average)	*p*-Value
KD26	238.47	0.0509	323.13	0.0190 *
KD31	244.40	0.0509	393.87	0.0006 ***
KD36	248.87	0.0509	360.53	0.0017 **
OE14	127.75	0.0611	341.88	0.0452 *
OE2	130.00	1.0000	244.75	1.0000
OE4	180.93	1.0000	344.53	0.6700
OE7	237.50	0.9312	503.50	0.0017 **
WT	194.73	1.0000	249.53	1.0000

The significant differences compared to the wild-type plants (WT) are denoted with ‘*’. The number of ‘*’ corresponds to the *p*-value: *p* < 0.001: “***”, *p* < 0.01: “**”, *p* < 0.05: “*”.

## Data Availability

The original contributions presented in this study are included in the article/supplementary material. Further inquiries can be directed to the corresponding author.

## Supplementary Materials

The following supporting information can be downloaded at: https://www.mdpi.com/article/10.3390/plants14223428/s1, Supplementary Figure S1: Genomic distribution and duplication patterns of GH5 genes in rice. Supplementary Figure S2: Multiple sequence alignment of rice GH5 domain sequences. Supplementary Figure S3: Expression profile of LOC_Os04g40510 based on RNA-seq database mining. Supplementary Figure S4: Germination rate of seeds from transgenic and wild-type plants at 3 DPI. Supplementary Figure S5: Phenotypic analysis of 14 DPI-old seedlings. Supplementary Figure S6: Overview of shoot growth, number of tillers, and number of panicles in transgenic lines and wild-type plants over time. Supplementary Figure S7: Seed weight analysis for the transgenic lines. Supplementary Data S1: Results and discussion data from phenotyping experiments of 2023. Supplementary Data S2: Statistical analysis of the chalkiness types. Supplementary Data S3: Database mining, sequence analysis, and primer design. Supplementary Data S4: Overview of number of plants grown per experiment.

## Abbreviations

The following abbreviations are used in this manuscript:

DPI	Days post imbibition
eGFP	Enhanced green fluorescent protein
GH	Glycosyl hydrolase
GH5	Glycosyl hydrolase family 5
GH5_x	Glycosyl hydrolase family 5 subfamily x
GSC	Grain surface crease
GUS	β-glucuronidase
KD	RNAi line/knock-down line
MWR	Milky-white rice
NB	Notched belly
NtP	*N*-terminal peptide
OE	Overexpression line
PR	Perfect rice
TIM	Triose-phosphate isomerase
WBR	White-belly rice
WCR	White-core rice
WPI	Weeks post imbibition
