# Supplementary material for: Characterization of a Rice GH5_11 Gene Associated with Endosperm and Seed Traits
Source: Plants (Basel). 2025 Nov 9;14(22):3428. doi: 10.3390/plants14223428 (PMC12656318; doi:10.3390/plants14223428)
Supplement: Supplementary file 1 [file plants-14-03428-s001.zip › Supplementary Data S1.pdf]

Results

1.1. Phenotypic analysis of transgenic lines (dataset 2023)

Shoot length of senescent transgenic lines and tiller number were counted for each plant. The plant height was not significantly affected for the transgenic lines compared to the wild-type plants (Figure 1). The knockdown line KD36 and the overexpression lines OE14 and OE7 had a higher number of tillers compared to wild-type plants. The number of tillers increased with 71.7%, 117.0% and 118.3% compared to the wild-type plants for the KD36, OE14 and OE7 lines, respectively.

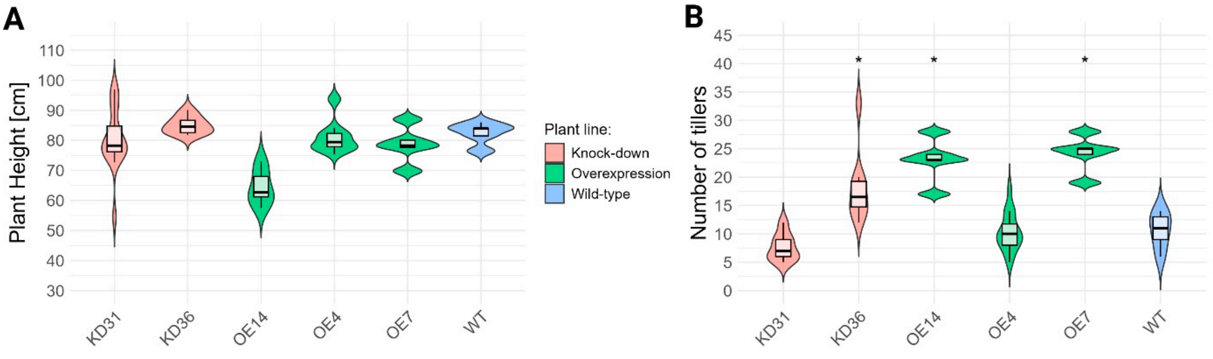

Figure 1. Vegetative characteristics of senescent transgenic plants (~20 WPI). (**A**) Plant height was measured for senescent (20 WPI) T3 plants. (**B**) The tiller number was counted for the same plants. Normality was evaluated through Shapiro-Wilk test and presence of homoscedasticity was determined by the Levene test. Depending on the results of the aforementioned tests, parametric (ANOVA followed by T-test) or non-parametric tests (Kruskal-Wallis followed by Wilcoxon rank sum test) were performed. Multiple hypothesis correction was performed with Benjamini-Hochberg. The significant differences compared to the wild-type plants (WT) are denoted with '*'. The number of '*' corresponds to the p-value: p<0.05:"*" .

Several agronomic traits were evaluated, such as the number of panicles, number of flowers per panicle, number of seeds per panicle and seed setting rate (Figure 2). Lines KD36, OE14 and OE7 had significantly higher numbers of panicles compared to the wild-type plants. Changes in the expression level of the *LOC_Os04g40510* gene does not affect the number of flowers per panicle but does affect the number of seeds within a panicle. Almost all the transgenic lines showed a reduced number of seeds per panicle relative to the wild-type plants with the exception of lines KD31 and OE4. The overall decrease in seeds per panicle was on average 12.3-49.8% for the knock-down lines and 28.7-93.1% for the overexpression lines.

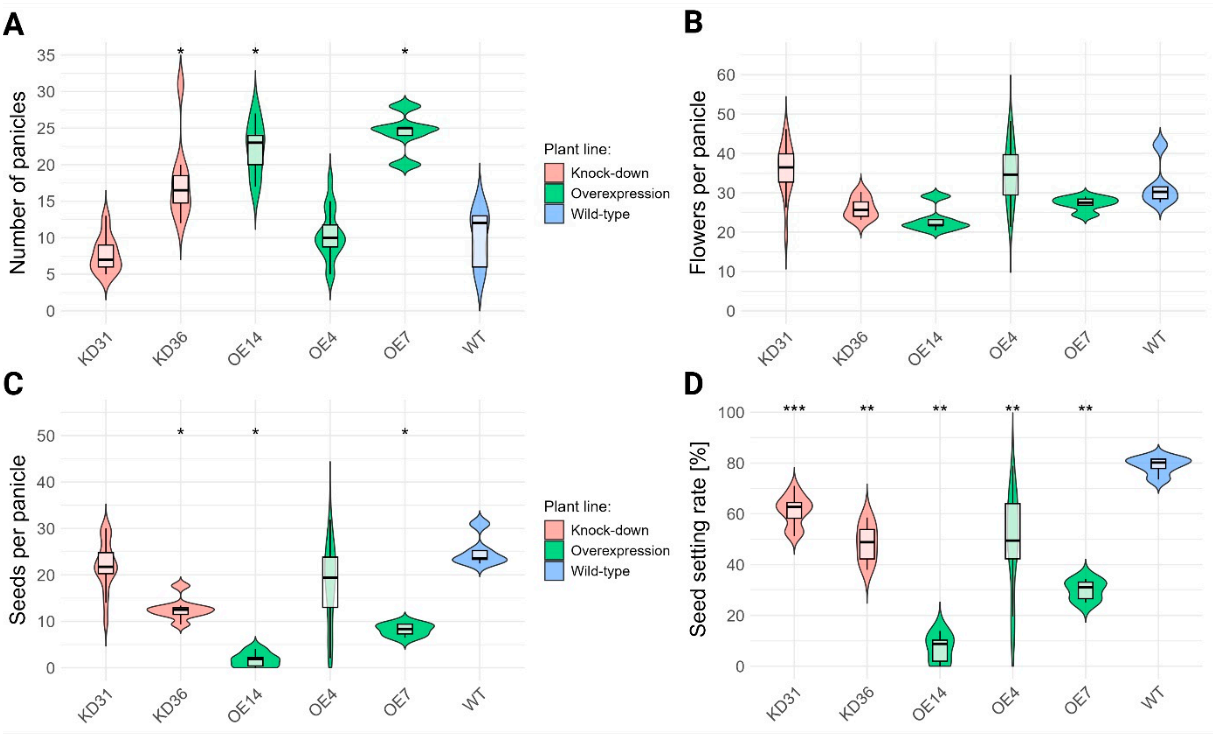

Figure 2. Generative characteristics for transgenic lines. (**A**) Number of panicles per plant was counted for 20 WPI-old T3 plants. (**B**) The number of flowers per panicle was counted. (**C**) The number of seeds per panicle was determined. (**D**) Based on the total number of seeds and the total number of flowers, the seed setting rate was calculated. Normality was evaluated through Shapiro-Wilk test and presence of homoscedasticity was determined by the Levene test. Non-parametric tests (Kruskal-Wallis followed by Wilcoxon rank sum test) were performed for all data given the absence of either normality and/or due to unequal variances. Multiple hypothesis correction was performed with Benjamini-Hochberg. The significant differences compared to the wild-type plants (WT) are denoted with '*'. The number of '*' corresponds to the p-value: p<0.0001:"****", p<0.001:"***", p<0.01 "**", p<0.05:"*".

The data is presented on a per-plant basis. Seed setting rate for a plant is the ratio of the total seed number over the total number of flowers. The total number of flowers per plant was estimated by the sum of the total number of seeds per plant and the total number of empty husks per plant. All transgenic lines had lower seed setting rates. The reduction of the seed setting was on average 22.5-39.0% for the knock-down lines relative to the wild-type plants. In the case of the overexpression lines a decrease of 38.0-91.2% was observed. This could be due to an overall increase in flowers per plant and/or due to an overall decrease of seeds per plant. The total number of seeds and flowers did not show any consistent significant trends with the wild-type plants (Table 1). Nonetheless, the overall number of seeds is lower for the overexpression lines compared to the wild-type plants, and the average number of flowers increased for the transgenic lines. In the latter case line KD31 is an exception.

Table 1. Summary of average number of seeds and flowers per plant and the significance compared to wild-type plants. Normality was evaluated through

Shapiro-Wilk test and presence of homoscedasticity was determined by the Levene test. Depending on the results of the aforementioned tests, parametric (ANOVA followed by T-test) or non-parametric tests (Kruskal-Wallis followed by Wilcoxon rank sum test) were performed. Multiple hypothesis correction was performed with Benjamini-Hochberg. The significant differences compared to the wild-type plants (WT) are denoted with '*'or '°'. The number of '*' corresponds to the p-value: p<0.0001:"****", p<0.001:"***", p<0.01 "**", p<0.05:"*" and p<0.1: "°".

Line	Total seed (average)	Total flower (average)
KD31	162.06	265.88
KD36	221.75	459.12
OE14	37.60 *	515.60 °
OE4	178.44	358.44
OE7	202.00	663.40 *
WT	245.40	310.60

1.2. Seed characteristics of the transgenic lines (2023)

For twenty seeds from each line (with five biological replicates) weight was measured (Figure 3). There is a significant decrease in seed weight for lines KD25, OE14 and OE7. There is a slight decrease in seed weight for lines KD36 and OE4 but this decrease was not significant.

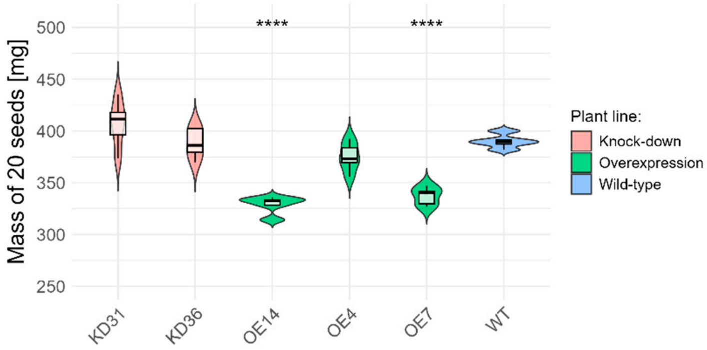

Figure 1~~3~~. Twenty randomly selected seeds of T3 plants were used to measure the mass (5 biological replicates). Normality was evaluated through Shapiro-Wilk test and presence of homoscedasticity was determined by the Levene test. Parametric tests (one-way ANOVA followed by two-sample t-tests) were performed for all data given normality and equal variances. Multiple hypothesis correction was performed with Benjamini-Hochberg. The significant differences compared to the wild-type plants (WT) are denoted with '*'. The number of '*' corresponds to the p-value: p<0.0001:"****", p<0.001:"***", p<0.01 "**", p<0.05:"*" .

Chalkiness was evaluated for approximately 100 seeds for each line (Figure 4). The percentage of seeds displaying the chalkiness phenotype is drastically increased in seeds from all transgenic lines relative to the wild-type plants. Approximately 70% of the seeds of the wild-type plants are completely translucent compared to 25-33% for the knock-down lines and 32-58% for the overexpression lines.

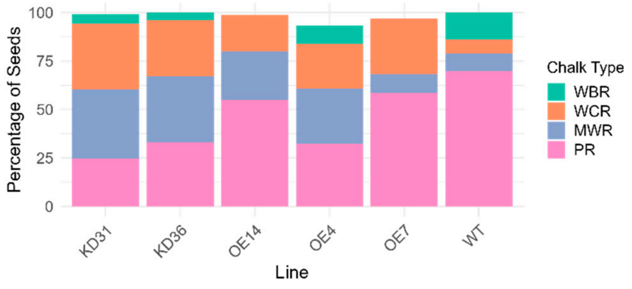

Figure 4. Stacked bar chart showing the average percentage composition of chalkiness types: perfect rice (PR), white-belly rice (WBR), white-core rice (WCR) and milky-white rice (MWR) across individual transgenic lines. Each bar represents the mean proportion of seeds exhibiting each chalkiness phenotype per line.

Conclusion

From the screening analysis, it was clear that some features are affected by the aberrant expression of *LOC_Os04g40510*, either in knock-down or overexpression lines. Tiller number and panicle number were increased after overexpression of *LOC_Os04g40510*, while the seed mass was reduced. The number of seeds per panicle and the seed setting rate were reduced for all the transgenic lines. In contrast, no significant effect was observed for yield. Moreover, the proportion of translucent (non-chalky) seeds was drastically reduced in the transgenic lines.

Material and methods

Plant materials

Oryza sativa subsp. japonica cv. Kitaake was used throughout the distinct experiments, as is described in the 'Material and methods'-section of the manuscript. T3-seeds were used for different transgenic lines to investigate distinct phenotypic features. The number of plants used for each transgenic line in the experiments is shown in Table 2.

Table 2. Overview of transgenic lines used in the phenotype screening experiment and the number of plants for each transgenic line.

Line	Number of plants
KD25	17
KD31	16
KD36	8
OE14	5
OE4	16
OE7	5
WT	5

Phenotypic analysis of rice plants (2023)

The phenotypes were evaluated for senescent plants approximately 20 weeks after imbibition (20 WPI). The plants were cultivated as described in the 'Material and methods' section of the manuscript.

Different metrics were determined including plant height, number of tillers, number of panicles, number of seeds, number of flowers and seed setting rate.

Seed characteristics of transgenic lines (2023)

The mass of 20 seeds was measured per line as one biological replicate. A total of five biological replicates were performed. In addition, the chalkiness of approximately 100 seeds was determined for each transgenic line. A transillumination plate was used to evaluate the different types of chalkiness as described by Yoshioka *et al.* (2007).

Statistical analysis was performed as described in the 'Material and methods'-section of the manuscript.
