# Supplementary material for: Characterization of a Rice GH5_11 Gene Associated with Endosperm and Seed Traits
Source: Plants (Basel). 2025 Nov 9;14(22):3428. doi: 10.3390/plants14223428 (PMC12656318; doi:10.3390/plants14223428)
Supplement: Supplementary file 1 [file plants-14-03428-s001.zip › Supplementary Figure S1.pdf]

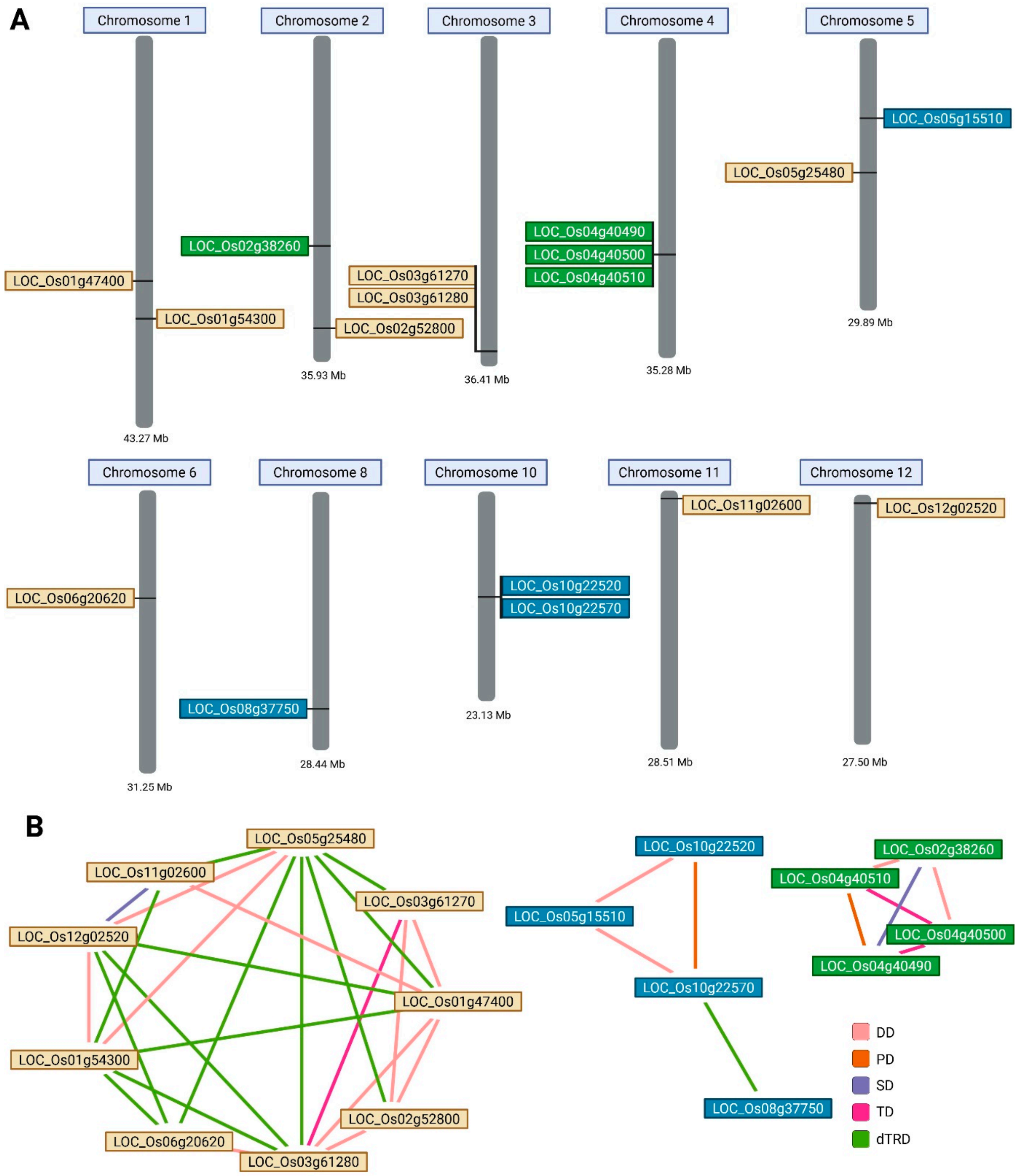

Supplementary Figure S1. Genomic distribution and duplication patterns of GH5 genes in rice. **(A)** GH5 sequences mapped on the Oryza sativa chromosomes. The size of the different chromosomes is indicated in Megabases (Mb). Sequence identifiers indicated on to the left and right of the chromosome correspond to the sequences localized on the positive and negative strand, respectively. Genes that are part of the GH5_7, GH5_11, and GH5_14 are displayed in yellow, green, and blue, respectively. **(B)** The different duplication events were evaluated for the different GH5 sequences and clustered using Cytoscape. The observed duplication events were dispersed duplications (DD), proximal duplications (PD), segmental duplications (SD), tandem duplications (TD), and DNA-transposed duplications (dTRD). The image was generated using BioRender.
