# Supplementary material for: Characterization of a Rice GH5_11 Gene Associated with Endosperm and Seed Traits
Source: Plants (Basel). 2025 Nov 9;14(22):3428. doi: 10.3390/plants14223428 (PMC12656318; doi:10.3390/plants14223428)
Supplement: Supplementary file 1 [file plants-14-03428-s001.zip › Supplementary Figure S2.pdf]

```
                       1                                      10
Loc_Os04g40510     ...........LACVNW...............................PSHLEPMLAEGL
Loc_Os01g47400_1   ..........................................YANGFNAYWLMLLAADPS
Loc_Os01g47400_2   ............................................NGFNAYWLMLLAADPS
Loc_Os01g54300     ..........................................YINGWNSYWLMDLAVEPN
Loc_Os02g38260     ...........LACVNW...............................PSHLEPVVTEGL
Loc_Os02g52800     .................F........................YSNGFNAYWLMYMASDPG
Loc_Os03g61270     ............................................................
Loc_Os03g61280     ..........................................FFSGFNAYWLMMMAADPA
Loc_Os04g40490     ...........LACANW...............................ASHLEPVAAEGL
Loc_Os04g40500     ...........LACANW...............................ASHLEPVAAEGL
Loc_Os05g15510     LPVRAVCLGGWLVTEGWILPSLFDAIPNKDLLDGLELQGEYQICNGYGTAKATPILRNHW
Loc_Os06g20620     .................L........................YVNGWNSYWLLP....AR
Loc_Os08g37750     ..VRAVNLGGWLVVEGWIKPSLFDGISNGN......LHGDYQLANGLGPDQAMVVLTEHR
Loc_Os10g22520     .PIRAVNLGGWLVTEGWILPSLFDDIPNKDLLDG..LQGEYQICNGYGKAKATQVLREHW
Loc_Os10g22570_1   .PIRSVNLGGWLVTEGWILPSLFDDIPNNDFLDG.KLQGEYQLCNGYGMKKATEVLREHW
Loc_Os10g22570_2   .PIRAVNLGGWLVTEGWIKPSLFDGISNKDLLDG..LQGEYQICNGYSAGNATEVLREHW
Loc_Os10g22570_3   .PIRSVNLGGWLVTEGWILPSLFDGIPNNDLLDG..PQGEYQICNGYGAEKASQVLREHW
Loc_Os11g02600     ...........................................IHGFNTYWLMSFAADQA
Loc_Os12g02520     ..........................................IIHGFNTYWLMSFAADQA
Loc_Os05g25480     .................F........................YVNGFNTYWLMVLAVDPS

                   20        30         40         50         60        70
Loc_Os04g40510     GKQPVGAIAKDVVAMGFNCVRLTWATFMVTN.ASYSSLTVAQSFQRLNLTESLAAIRVNN
Loc_Os01g47400_1   QRGKVSAALGEAAGHGLTVART....WAFSDGGGGNAL...................QLS
Loc_Os01g47400_2   QRGKVSAALGEAAGHGLTVART....WAFSDGGGGNAL...................QLS
Loc_Os01g54300     TRPRVSSMFRTAVSMGLTVCRT....WAFND.GSYNAL...................QLS
Loc_Os02g38260     GMQPVDAISKKVASLGFNCVRLTYPIALATN.ASLSSLTVRRSLLAHGLAGAVAGVEANN
Loc_Os02g52800     DRSKAAGVLQQAASLRATLVRT....WAFSD.GGYRPL...................QKS
Loc_Os03g61270     ............................................................
Loc_Os03g61280     LRGAVATAFQQASAHGLNLART....WAFSD.GGDQPL...................QSS
Loc_Os04g40490     SRRGLGDIAARVAAMGLNCVRLTWPTYLATN.ATLANLPLRASLERLGMPESVAGVRVNN
Loc_Os04g40500     SRRGVGDIAARVAAMGFNCVRLTWPTYLATN.ATLASLPLRWSLERFGMRESVAGVRVNN
Loc_Os05g15510     STYIVEDDFKFISASGLTAVRIPVGWWIASD..........................PNP
Loc_Os06g20620     SPALAAEMLRRGRRMGLSVCRT....WAFSD.GGPGAL...................QIS
Loc_Os08g37750     KNFITGKDFYFLSKNGINAVRIPVGWWIAYD..........................PNP
Loc_Os10g22520     RTYIVESDFKFISTSGLNAVRIPVGWWIASD..........................PNP
Loc_Os10g22570_1   STYILENDFKFISSNGLNAVRIPVGWWIASD..........................PNP
Loc_Os10g22570_2   NTFIVEDDFKFISSNGLNAVRIPVGWWIASD..........................PNP
Loc_Os10g22570_3   STYIVESDFEFISSSGLNAVRIPVGWWIASD..........................PNP
Loc_Os11g02600     TRLRVTAAI...AEAGLNVCCT....WAFSD.GGYRAL...................QTA
Loc_Os12g02520     TRPRVTAAIAEAAEAGLNVCRT....WAFSD.GGYRAL...................QTV
Loc_Os05g25480     TRGKVTEVFRQAAAVGLTVCRT....WAFND.GGWRAL...................QKS

                     80       90                         100       110
Loc_Os04g40510     PSLVDLKLIDAFKAVVSSL..............GENGVMVILDNHVSKPG..........
Loc_Os01g47400_1   PGNYNENTFKGLDFVLSEA..............RKYGIKVILSL.......VDNYDSFGG
Loc_Os01g47400_2   PGNYNENTFKGLDFVLSEA..............RKYGIKVILSL.......VDNYDSFGG
Loc_Os01g54300     PGHFDERVFKALDRVVAEA..............SEHGVRLILSL.......ANNLDAYGG
Loc_Os02g38260     PGLLDLTLIESFRAVVDSL..............GESGVMVILDNHVSRPG..........
Loc_Os02g52800     PGVYNEDMFMGLDFVIAEA..............KKRGLYLILSL.......VNNWDGFGG
Loc_Os03g61270     ............................................................
Loc_Os03g61280     PGVYNETMFQGLDFVIAEA..............RRHGIYLLLCL.......TNNFDNFGG
Loc_Os04g40490     PGLLDLPLIDVFQEVVSAL..............AKNNIMVILDNQMTTPG..........
Loc_Os04g40500     PGLLDLPLIDVFQEVVSAL..............ARNNIMVILDNQMTTPG..........
Loc_Os05g15510     PAPYVGGSLQTLDNAFKWA..............EKYKLGVIIDLHAA.PGSQNPWEHSSS
Loc_Os06g20620     PGRFSEAVFQVLDYVIYEA..............RRNHIRLILCL.......VNNLDNLGG
Loc_Os08g37750     PAPFVSGSLDTLDRAFYWAHFKSAQYGGSDFDIRIYGLKCIIDLHAA.PGSQNGMEHSAS
Loc_Os10g22520     PAPFVGGSLQALDNAFKWA..............EKYNLGVIVDLHAA.PGSQNPFEHSAS
Loc_Os10g22570_1   PAPFVGGSLEALDNAFRWA..............EKYNLGVIVDLHAA.PGSQNPWEHSGS
Loc_Os10g22570_2   PAPFVGGSLQALDNAFKWA..............EKYNIGIIVDLHAA.PGSQNRLDHSAS
Loc_Os10g22570_3   PAPFVGGSLQALDNAFKWA..............ENYNIGVIVDLHAA.PGSQNHWEHSAT
Loc_Os11g02600     PFHYDEDVFRALDFVVSEA..............RRHNMRLILSL.......CNNWEDYGG
Loc_Os12g02520     PFHYDEDVFQALDFVVSEA..............KRHNMRLILSL.......CNNWEDYGG
Loc_Os05g25480     PGVYDEEVFKALDFVVSEA..............RKHKIRLILPL.......INNWDDYGG
```

```
                      120                 130          140       150
Loc_Os04g40510     .....WCCGNNDG.....NGFFGD....AYFDP...DVWVDGLTKMATMFAAVPSVVAM
Loc_Os01g47400_1   RKQYVNWARAQGQGI.GSDDEFFTNPVVKGFYKNHVKTVLTRKNTITGVAYRDDPTILAW
Loc_Os01g47400_2   RKQYVNWARAQGQGI.GSDDEFFTNPVVKGFYKNHVKTVLTRKNTITGVAYRDDPTILAW
Loc_Os01g54300     KRQYYRWAWEEGVGLTASNDSFFFDPAIRDYFKVYLKTLLMRKNHLTGLEYRDDPTILAW
Loc_Os02g38260     .....WCCADDDG.....NGFFGD....RHFDP...DAWVRGLGAMAALFAGVPNVVGM
Loc_Os02g52800     KKQYVQWARDQGHNL.GSDDDFFRSDVTKQFYKNH..AVLTRVNKITGVAYKDDPTIFAW
Loc_Os03g61270     ............................................................
Loc_Os03g61280     KRQYYRWAGDAGHNL.TSDDDFFTSTIVKSYFKNHVKTVLTRVNTLTGVAYKDDPTIFAW
Loc_Os04g40490     .....WCCSENDG.....NGFFGD....KYFGP...EEWLKGLSAMATMFRNTKNVVGM
Loc_Os04g40500     .....WCCSTTDG.....NGFFGD....KYFDP...EEWLNGLKTMATMFRKTKNVVGM
Loc_Os05g15510     RDGTQEWGTSDANI.......................AETVQVIDFLASRYAKSPSLLAV
Loc_Os06g20620     KAQYVQWAQAAGANMTNSTDSFYSHPTIKRYYKDYVKAILTRRNSYSRIRYSDEPAIFAW
Loc_Os08g37750     RDGSVDW.PSPANI.......................EKTLDVINFLAQRYANNPSLLGI
Loc_Os10g22520     RDGSQDWGTTDANI.......................AQTVQVIDFLTHRYASSPSLLAV
Loc_Os10g22570_1   RDGSQTWGTTDETI.......................IQTVQVIDFLASRYAKSPSLLAV
Loc_Os10g22570_2   RDGSLEWGTSAANI.......................AQTVGVIDFLASRYAKSSSLLAI
Loc_Os10g22570_3   RDGSLEWGTTDTSI.......................TQTVQIIDFLASRYANSPSLLAI
Loc_Os11g02600     KAQYVRWGKEAGLDL.TSEDDFFSDPTIKSYYKAFVEAVVTRINTVTNETYKDDPTILAW
Loc_Os12g02520     KAQYVRWGKEAGLDL.TSEDDFFSDPTIKSYYKAFVEAVVTRINTVTNETYKDDPTILAW
Loc_Os05g25480     KAQYVRWAQAAAAG..AGADAFFSDETVRGYFKSHVTAVLTRVNAYTGVAYRDDPTIMAW
```

```
                     160       170        180        190
Loc_Os04g40510     SLRNELRGPRQNSA.DWYKYMQRGAEAVHAAN.PRVVVILSGMSF...............
Loc_Os01g47400_1   ELMNEPRCQSDLSGRTVQSWITEMAAHVKSID.RNHMLEVGLEGFYGASSPSRIAAVNPS
Loc_Os01g47400_2   ELMNEPRCQSDLSGRTVQSWITEMAAHVKSID.RNHMLEVGLEGFYGASSPSRIAAVNPS
Loc_Os01g54300     ELMNEPRCTSDPSGDTLQRWMEEMSAYVKSID.KKHLLTVGTEGFYGPTSSQEKLNINPG
Loc_Os02g38260     SLRNELRGPRQNAD.DWYRYMQMGAEAVHAAN.PAALVIMGGLGY...............
Loc_Os02g52800     ELINEPRCQSDLSGKTLQAWVTEMAGYVKSVD.PNHMVEIGLEGFYGESMHKN...FNPG
Loc_Os03g61270     ..MNEPRCDADPTGGMVQAWVEEMAPYVKRVDGGRHLVTAGLEGFYGDGEHES.KELNPW
Loc_Os03g61280     ELMNEPRCYADPTGAMVQAWVEEMAPYVKSVD.GRHLVTPGLEGFYGAGEHES.KELNPW
Loc_Os04g40490     SLRNELRGS.KNVS.LWFRYMQLGAEVVHAAN.PGVLVILSGLNF...............
Loc_Os04g40500     SLRNELRGPYENVS.LWYRYMKEGAESVHTAN.PDVLVILSGLEF...............
Loc_Os05g15510     ELMNEPFAPRATLE.SLMKYYHDGYNAVRKYS.STAYVIMSNRLG...............
Loc_Os06g20620     ELMNEPRCVSNSSGPYLQAWIAEMAAYVKSLD.TNHLVTVGTEGFYGPGIAER.LGVNPG
Loc_Os08g37750     ELLNEPSAGAVPLG.TLVSYYKTGYQIVRSYS.DKAYVIFCQRIG...............
Loc_Os10g22520     ELLNEPLAPGVTLP.ALMRYYKDGYNAVRKYT.STAYVVMSNRLS...............
Loc_Os10g22570_1   ELLNEPLAPKVSAG.MLKKYYQDAYNAVRKYT.SDAYVIMSNPIN...............
Loc_Os10g22570_2   ELLNEPLAPDVPVD.TLTKYYQDAYNAVRKYT.LQAYVILSTRMS...............
Loc_Os10g22570_3   ELLNEPWGPDVPLE.KLKKYYEDAYNVVRKYT.AKAYVIMSNRLA...............
Loc_Os11g02600     ELINEPRCPSDPSGDTLQVFITYV....................FFG.............
Loc_Os12g02520     ELINEPRCPSDPSGDTLQAWIEEMASYVKSID.PVHLLEIGIEGFYGLSTPEL.LPVNPD
Loc_Os05g25480     ELMNEPRCASDPTGDTLQAWIAEMAFHVKSVD.PAHLLGVGAEGFYGPSSPPARLRVNPN
```

```
                        200       210            220       230       240
Loc_Os04g40510     .....DNDLAFLNSRQV.NVSFA....GKVAFEVHWYGFSDGQAWRAGNANQVCARVAAS
Loc_Os01g47400_1   G...YQLGTDFIANNQVPGIDFA....TVHSYPDQWLS........GKDDQAQLGFMGRW
Loc_Os01g47400_2   G...YQLGTDFIANNQVPGIDFA....TVHSYPDQWLS........GKDDQAQLGFMGRW
Loc_Os01g54300     EWFPNNYGADFIRNSKIQDIDFA....SVHVYPDNWLQ........HASLDEKLKFMTRW
Loc_Os02g38260     .....DTDLSFLAARPV.DVSFAAAERGKLVFELHWYSFADARAWESEDANEVCGRVARG
Loc_Os02g52800     ....YTVGTDFIANNLVPAVDFA....TIHSYPDQWVS........GASSDEQVAFMRKW
Loc_Os03g61270     G...IYYGTNYVATHRAAGVDFA....TIHLYPDVWLW........GSTADEQAAFFRNW
Loc_Os03g61280     G...IYYGTNYVATHRTAAVDFA....TIHLYPDVWLW........GSSADEQATFFRNW
Loc_Os04g40490     .....DNTLDFLVPNQI.QLTFT....GKLVFEQHWYGFSDDGNWGSQNQNDACGMVVDS
Loc_Os04g40500     .....DNTLNFVVPNQI.HLSFT....GKLVFEQHWYGFSDGGNWESQNQNDVCGMVVGF
Loc_Os05g15510     ....PHDPTEFL........QFA.NGFPRAVIDVHYYTVFN.DLFNNLTVQQNIDFIKSN
Loc_Os06g20620     EWA.ASLCSDFIQNSAVEHIDFA....SVHAYPDSWLP........RASLEEKVRYLSNW
Loc_Os08g37750     ....NADPMELY........QAD.LGPTNTVVDLHYYNLFD.PFFEKLNATENIQFIYNN
Loc_Os10g22520     ....AS.NTELL........GFA.AGFPGAVLDVHYYNLFT.SSFNGLTVDQNIDYVRTN
Loc_Os10g22570_1   ....ADYSNEIL........QFA.GGFFGAVFDVHYYNMFN.GSFDNTTAEWNIQFVRND
Loc_Os10g22570_2   ....GD.PTEFL........SVA.SSLFGAVIDVHYYNLYN.SMFDNYTVEQNINFVRNN
Loc_Os10g22570_3   ....GESNTELL........DFA.SRFPGVVIDVHYYNLFNDDTFKNLNVEQNIEFVKNS
Loc_Os11g02600     ............................................................
Loc_Os12g02520     EYS.GHAGTDFIRNHQAPGIDLA....SIHVYSDTWLP........HSIKENHLQFVDKW
Loc_Os05g25480     ADV.ALAGADFVRNHRVLGVDFA....SVHVYPDTWLP.......AGATKEAQLRFATSW
```

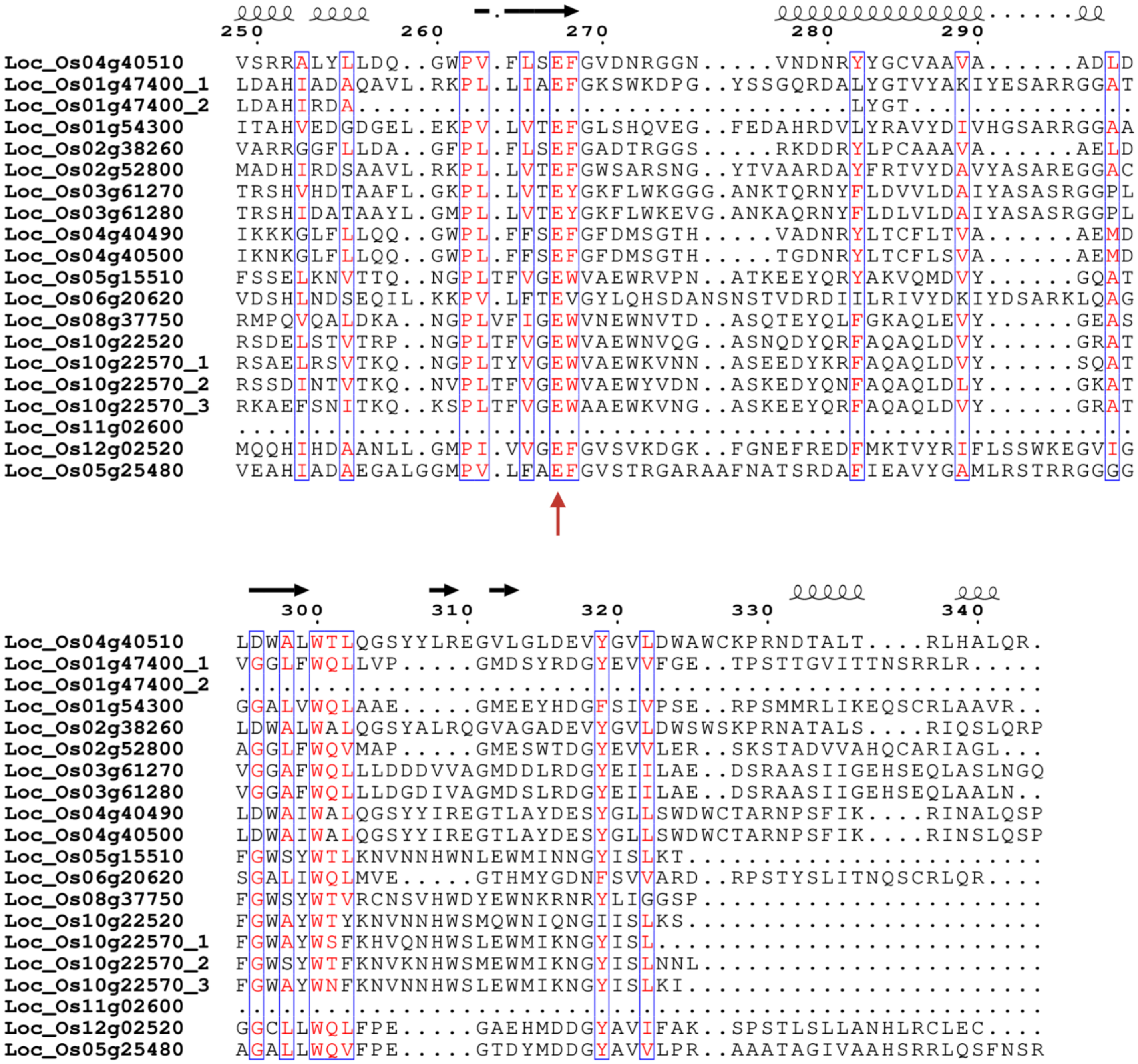

Supplementary Figure S2. Multiple sequence alignment of rice GH5 domain sequences. Identical residues across all sequences are highlighted in red boxes, while chemically similar residues are shown as red letters within blue boxes. Red arrows below the alignment indicate the catalytic glutamate residues. Predicted secondary structure elements based on the GH5 domain of LOC_Os04g40510 are shown above the alignment, with black arrows representing β-strands and black curls indicating α-helices.
