# Supplementary material for: Characterization of a Rice GH5_11 Gene Associated with Endosperm and Seed Traits
Source: Plants (Basel). 2025 Nov 9;14(22):3428. doi: 10.3390/plants14223428 (PMC12656318; doi:10.3390/plants14223428)
Supplement: Supplementary file 1 [file plants-14-03428-s001.zip › Supplementary Figure S3.pdf]

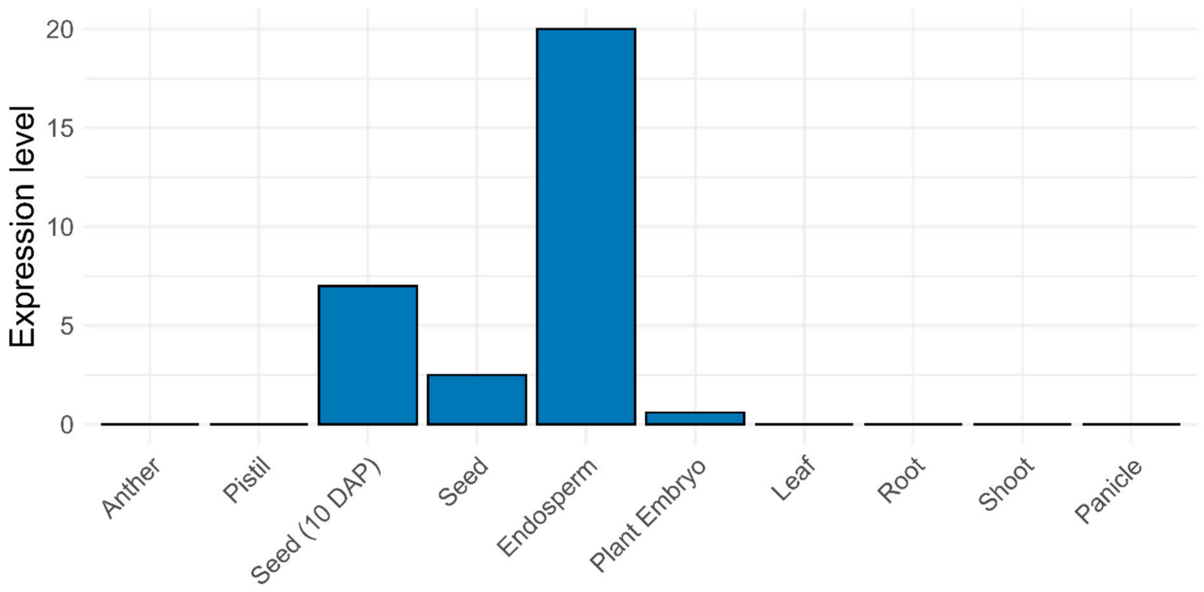

Supplementary Figure S3. Expression profile of *LOC_Os04g40510* based on RNA-seq database mining (Expression Atlas). The expression levels are shown in transcripts per million (TPM) across different developmental stages and tissues. For each tissue, values represent averaged normalized expression data provided by the database. Multiple datasets from independent studies were available for seed-related samples, allowing calculation of mean TPM values. Replicate-level data were not accessible; therefore, no statistical testing or variance estimates could be applied. DAP, days after pollination.
