# Supplementary material for: Characterization of a Rice GH5_11 Gene Associated with Endosperm and Seed Traits
Source: Plants (Basel). 2025 Nov 9;14(22):3428. doi: 10.3390/plants14223428 (PMC12656318; doi:10.3390/plants14223428)
Supplement: Supplementary file 1 [file plants-14-03428-s001.zip › Supplementary figure S4.pdf]

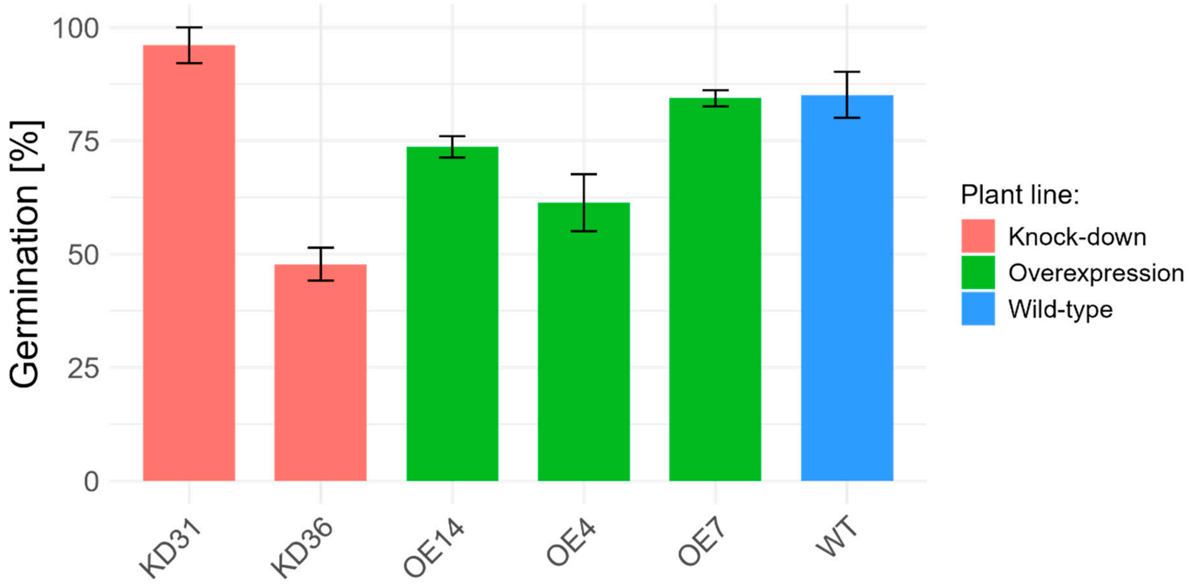

Supplementary Figure S4. Germination rate of seeds from transgenic and wild-type plants on non-selective MS-plates at 3 DPI. The mean was determined from four biological replicates each containing approximately 10 seeds. The error bars represent the standard error. Normality was evaluated through Shapiro-Wilk test and the presence of homoscedasticity was determined by the Levene test. Non -parametric tests Kruskal–Wallis test was applied, followed by pairwise Wilcoxon rank sum test, comparing the transgenic lines with the wild-type. Multiple hypothesis correction was performed with Benjamini-Hochberg. Germination data for line KD26 are not shown due to limited seed availability. No significant differences were observed among the lines ($p > 0.05$); the lowest p-value was obtained for line KD36 ($p = 0.088$).
