# Supplementary material for: Characterization of a Rice GH5_11 Gene Associated with Endosperm and Seed Traits
Source: Plants (Basel). 2025 Nov 9;14(22):3428. doi: 10.3390/plants14223428 (PMC12656318; doi:10.3390/plants14223428)
Supplement: Supplementary file 1 [file plants-14-03428-s001.zip › Supplementary Figure S5.pdf]

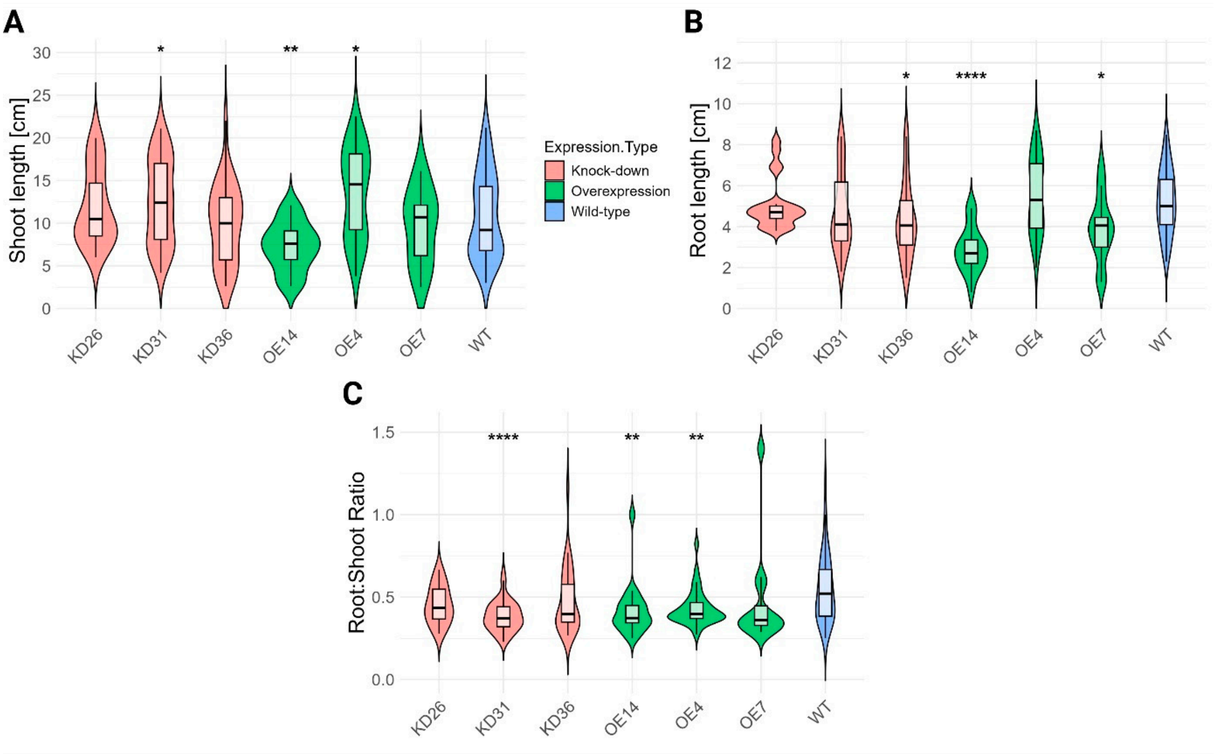

Supplementary Figure S5. Phenotypic analysis of 14 DPI -old seedlings. (**A**) Shoot length, (**B**) root length, and (**C**) root-to-shoot ratio were determined. Normality was evaluated through Shapiro-Wilk test and presence of homoscedasticity was determined by the Levene test. Depending on the results of the aforementioned tests, parametric (ANOVA followed by T-test) or non-parametric tests (Kruskal-Wallis followed by Wilcoxon rank sum test) were performed. Multiple hypothesis correction was performed with Benjamini-Hochberg. The significant differences compared to the wild-type (WT) are denoted with '*'. The number of '*' corresponds to the p-value: p<0.0001:"****", p<0.01 "**", p<0.05:"*" .
