# Supplementary material for: Characterization of a Rice GH5_11 Gene Associated with Endosperm and Seed Traits
Source: Plants (Basel). 2025 Nov 9;14(22):3428. doi: 10.3390/plants14223428 (PMC12656318; doi:10.3390/plants14223428)
Supplement: Supplementary file 1 [file plants-14-03428-s001.zip › Supplementary figure S6.pdf]

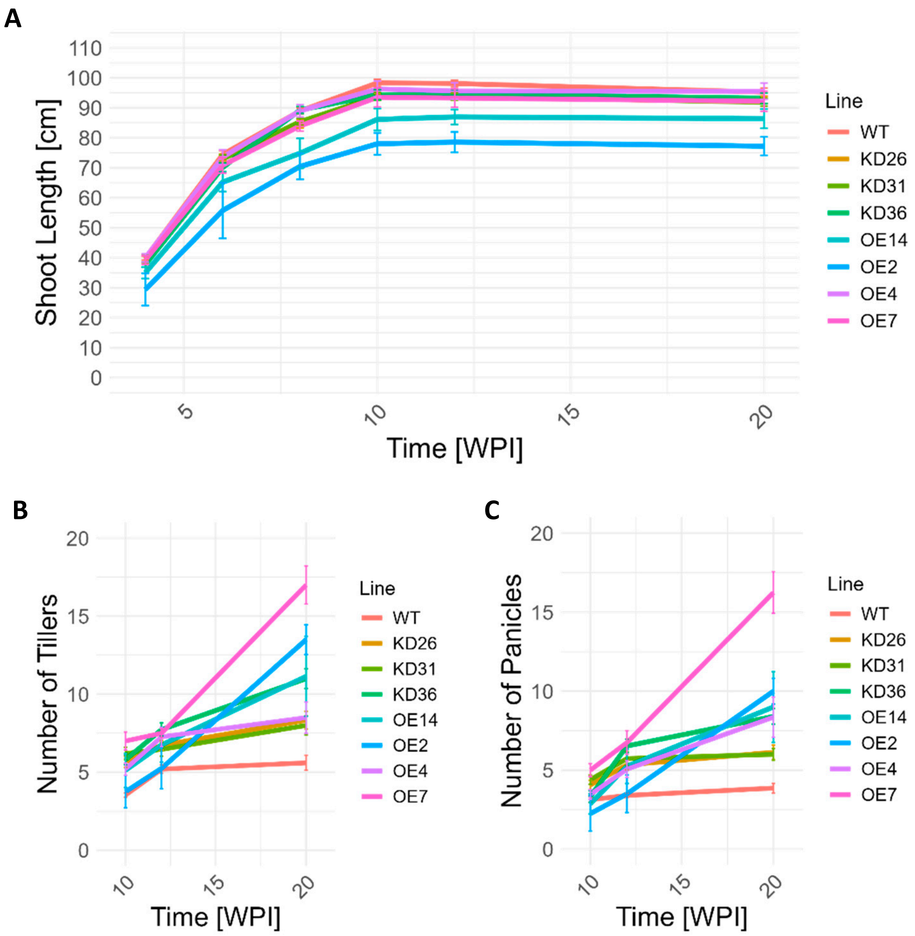

D

Line	p-value growth	p-value tillers	p-value panicles
WT	1.0000	1.0000	1.0000
KD26	0.0983	0.0011	0.0003
KD31	0.0711	0.0290	0.0003
KD36	0.1780	0.0002	0.0001
OE2	0.0099	0.0425	0.0296
OE4	1.0000	0.0057	0.0040
OE7	0.1102	0.4112	0.3419
OE14	0.0023	0.1251	0.0330

Supplementary Figure S6. Overview of (**A**) shoot growth, (**B**) number of tillers and (**C**) number of panicles in transgenic lines and wild-type plants over time (indicated as weeks after imbibition (WPI)). The error bars represent the standard error. (**D**) Statistics were done using the area under the curve due to the non-normality and heteroscedasticity of the data. p-values were adjusted using Benjamini-Hochberg and are highlighted in orange when lower than 0.05.
