# Supplementary material for: Characterization of a Rice GH5_11 Gene Associated with Endosperm and Seed Traits
Source: Plants (Basel). 2025 Nov 9;14(22):3428. doi: 10.3390/plants14223428 (PMC12656318; doi:10.3390/plants14223428)
Supplement: Supplementary file 1 [file plants-14-03428-s001.zip › Supplementary Figure S7.pdf]

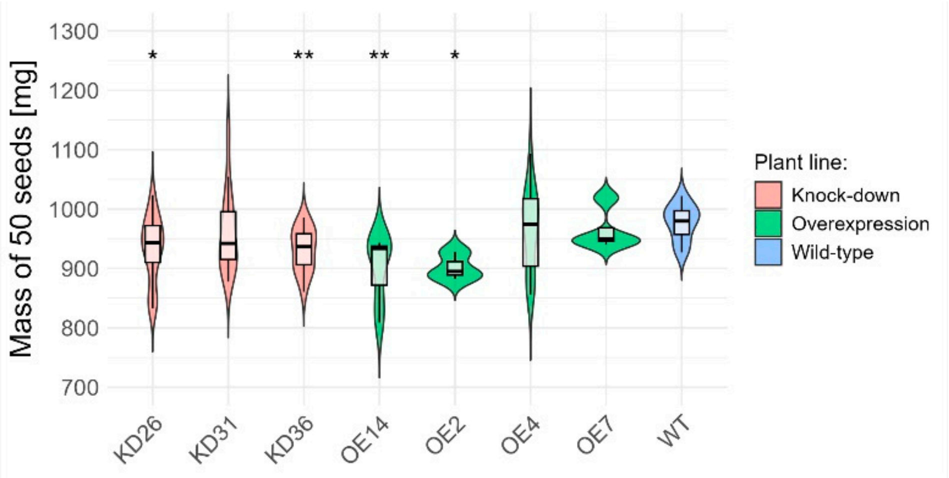

Supplementary Figure S7. 50 seeds of each plant were used to measure the seed weight for T3 plants. Normality was evaluated through Shapiro-Wilk test and presence of homoscedasticity was determined by the Levene test. Non-parametric tests (Kruskal-Wallis followed by Wilcoxon rank sum test) were performed for all data given the absence of either normality and/or due to unequal variances. Multiple hypothesis correction was performed with Benjamini-Hochberg. The significant differences compared to the wild-type (WT) are denoted with '*'. The number of '*' corresponds to the p-value: p<0.01 "**", p<0.05:"*".
